# Metal Peptide Conjugates in Cell and Tissue Imaging and Biosensing

**DOI:** 10.1007/s41061-022-00384-8

**Published:** 2022-06-15

**Authors:** Karmel S. Gkika, David Cullinane, Tia E. Keyes

**Affiliations:** School of Chemical Sciences, National Centre for Sensor Research, Dublin City University, Dublin 9, Ireland

**Keywords:** Transition metal luminophores, Photophysics, Imaging, Metal conjugates, Peptide, Imaging, Cell-penetrating peptides, Signal peptides, Octaarginine, MPP

## Abstract

Metal complex luminophores have seen dramatic expansion in application as imaging probes over the past decade. This has been enabled by growing understanding of methods to promote their cell permeation and intracellular targeting. Amongst the successful approaches that have been applied in this regard is peptide-facilitated delivery. Cell-permeating or signal peptides can be readily conjugated to metal complex luminophores and have shown excellent response in carrying such cargo through the cell membrane. In this article, we describe the rationale behind applying metal complexes as probes and sensors in cell imaging and outline the advantages to be gained by applying peptides as the carrier for complex luminophores. We describe some of the progress that has been made in applying peptides in metal complex peptide-driven conjugates as a strategy for cell permeation and targeting of transition metal luminophores. Finally, we provide key examples of their application and outline areas for future progress.

## Introduction

Fluorescence microscopy is one of the most important and ubiquitous tools in the life sciences. Its applications vary from the simple visualisation of fixed samples to quantitative and dynamic determination of biological processes in living cells and tissues. Luminescent metal complexes are emerging as highly useful probes for fluorescence microscopy, competing with more traditional organic fluorophores due to their excellent, tuneable photophysical properties and their amenity to sensing applications.

Indeed, many metal complex luminophores are addressable through multimodal methods and offer prospects for applications in imaging, sensing and theranostics.

Probes with visible to near-infrared (NIR) excitation and emission are required in bioimaging. In particular, emission that coincides with the biological optical window (650–1000 nm) is preferable because NIR light is more isotropically scattered by tissue, and light in this frequency range is not absorbed by biomolecules. It is therefore more penetrative through biological tissue and moreover autofluorescence from endogenous sources upon NIR excitation is minimal. As luminescence from most transition metal complexes is formally phosphorescence, their emission exhibits a large Stokes shift (energy difference between absorption and emission maxima). This is advantageous as it avoids artefactual effects from inner filter effects or self-quenching, which may be more prevalent when the probe is localised at high concentrations. Another rarely considered advantage of the large Stokes shift is that it facilitates dual use of such complexes as probes in tandem luminescence and resonance Raman measurements under resonant excitation, since the Stokes shift enables excitation and detection of the resonance Raman signature away from the overwhelming emission signature [1, 2]. The long-lived and triplet nature of the excited state of many metal complex luminophores, notably those of ruthenium(II) and iridium(III), render them susceptible to quenching by analytes such as molecular oxygen (O_2_), reactive redox species or pH. The characteristic luminescence lifetime- or intensity-based response typically reflects the interaction of the metal complex with these species within a cellular or tissue environment.

Luminescence intensity-based sensing can be performed using conventional instrumentation such as a fluorescence microscope or plate reader. An important limitation is that the absolute signal intensity alone, cannot typically be used as a reliable quantitative marker for a single target analyte because intensity *in cellulo* is influenced by many factors. Most notably, it will vary with distribution in the cell which is rarely uniform, and physiochemical issues such as photodamage, probe leaching and interaction with species such as proteins or lipid membranes within the cellular environment can influence emission intensity. Additionally, intensity can be affected by the excitation source or detector drift and sensitivity. A practical approach to facilitate use of emission intensity for sensing is to apply *ratiometric sensing.* The ratiometric approach involves referencing the sensor probe emission signal to a stable emission signal from a dye that does not respond to the analyte or species of interest, but is subject to the same instrumental fluctuations that influence the intensity of the analytical signal.

An alternative way to obtain insight into a particular analyte that is not dependent on dye concentration or instrumental fluctuations is to apply fluorescence lifetime imaging microscopy (FLIM), *or phosphorescence lifetime in the case of most metal luminophores*. It is a quantitative imaging technique that can be used for real-time mapping of the cellular and tissue microenvironment, including cell functions and metabolic changes where the lifetime of a fluorophore is influenced by its local environment. As indicated, unlike intensity-based methods such as confocal fluorescence imaging, the image is independent of luminophore concentration, reflecting only the emission lifetime distribution of the probe.

A challenge that has traditionally impeded the application of metal complexes as bioimaging and biosensing probes is the poor uptake of such materials into cells. This has been widely overcome in recent years with a variety of strategies involving modification of the physicochemical properties of the complex or bioconjugation [3]. Conjugation of complexes to cell-penetrating and signal peptides specifically, has proven to be a particularly attractive and reliable method for achieving efficient cellular uptake without the use of permeabilisation agents. In particular, in the context of metal complex luminophores, this approach has the potential to very specifically drive the probe to target organelles with complex membrane structures such as the mitochondria or nucleus.

This review focuses on the more commonly studied luminescent transition row complexes of Ru(II), Ir(III), Os(II) and Re(I) with some examples from less well studied transition metal complexes such as Pt(II), Pd(II), Rh(III) and Zn(II).

## Photophysical Profile of an Ideal Chromophore for Bioimaging

Luminescence imaging, including, particularly, confocal fluorescence and luminescence lifetime imaging methods, are widely used techniques in biochemistry and molecular biology as they offer high contrast, sensitivity, good resolution and flexibility in choice of luminophore probe. In addition, with commercialisation of more advanced imaging methods, including super-resolution and multiphoton methods, there is a growing need for probes that meet the demands of these methods, including robust photostability, sensitive environmental responsivity, high membrane permeability and targeted localisation. Indeed, studies to date have demonstrated that metal complexes can be applied in interrogating the cell environment and studying dynamic processes in vivo via a variety of imaging methods and that they have the synthetic versatility to tune to the desirable photophysical properties while maintaining biocompatibility and low cytotoxicity.

### Favourable Properties of Metal Complexes

The ideal photophysical/optical characteristics of a luminescent imaging probe vary depending on the imaging methodology, although a number of characteristics are common to all, including the need for high molecular brightness (product of the molar extinction coefficient and quantum yield) and photostability. A diverse range of probes have been developed for fluorescence/luminescence imaging, including fluorescent proteins, expressed in situ in the cell, or exogenously applied probes, including organic fluorophores, nanoparticles, quantum dots and metal complexes. Organic fluorophores such as rhodamine, cyanine dyes, and the Alexa Fluor and Atto dyes, have been used widely as contrast agents in fluorescence microscopy to date as they exhibit high molecular brightness and in the case of Atto and Alexa Fluor probes, show good photostability. However, intrinsic drawbacks of organic fluorophores include a narrow Stokes shift which leads to inner filter effects and self-quenching at high optical densities, and in many cases, limited photostability. They also frequently show poor solubility in aqueous media, and so, application in cells often requires pre-dissolution in organic solvent that promotes cellular permeation but often through damage to the membrane. Finally, the short emission lifetime of organic fluorophores (usually in the range of 1–5 ns) is typically too short to enable time gating as a method to discriminate probe emission from background autofluorescence, and in sensing applications, limits quenching capability for diffusing species (the dye singlet states limit oxygen sensing also).

**Fig. 1:**
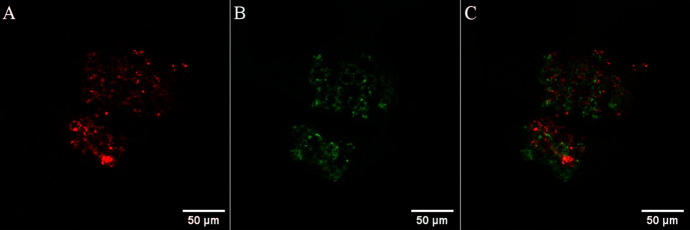
Confocal imaging of a single live human pancreatic cancer (HPAC) spheroid treated with an Os(II) polyarginine probe, [Os-(R_4_)_2_]^10+^ at 100 μM/48 h. Using a 490 nm white light laser for excitation, emission was collected between **A** 650 and 800 nm; Os(II) channel and **B** 500–570 nm; auto-fluorescence window. **C** Os(II)/autofluorescence channel overlay. Reprinted (adapted) with permission from Ref. [10] (https://pubs.acs.org/doi/10.1021/acs.inorgchem.1c00769). Further permissions related to the material excerpted should be directed to the ACS

Aside from time gating, another approach to avoid autofluorescence interference in cellular or tissue imaging is to use a probe that emits in the red or NIR spectral range. Autofluorescence, excited at short excitation wavelengths, occurs from naturally fluorescent molecules within the cell and tissue environment or medium and usually decays on the nanosecond timescale. Nicotinamide adenine dinucleotide (NAD) and flavin adenine dinucleotide (FAD) are examples of intrinsic biological fluorophores for which several studies on their fluorescent properties have been carried out. Even if, for example, in Stokes-shifted emission, the probe is excited in the blue visible spectral range, which excites autofluorescence, its detection can be avoided if the probe emission is in the red region. In the context of luminescence imaging, but also therapy, a probe absorbing in the low-energy visible or NIR region is also desirable as this allows for deeper light-tissue penetration and avoids biological damage from continuous photo-irradiation into spectral regions where there is absorbance by tissue [4–9]. This is illustrated in Fig. 1 where emission from an Os(II) polypyridyl complex in the 650–800-nm region avoids any significant background signal from biological autofluorescence of a multicellular spheroid [10].

Transition metal complexes have also shown good photostability, which is particularly robust in the case of osmium(II) polypyridyl luminophores, where photodecomposition and photobleaching can be completely avoided. To date, the coordination compounds of the (second and third row) d^6^ metals Ru(II), Os(II) or Ir(III) are amongst the most widely studied transition metal imaging probes.

**Fig. 2:**
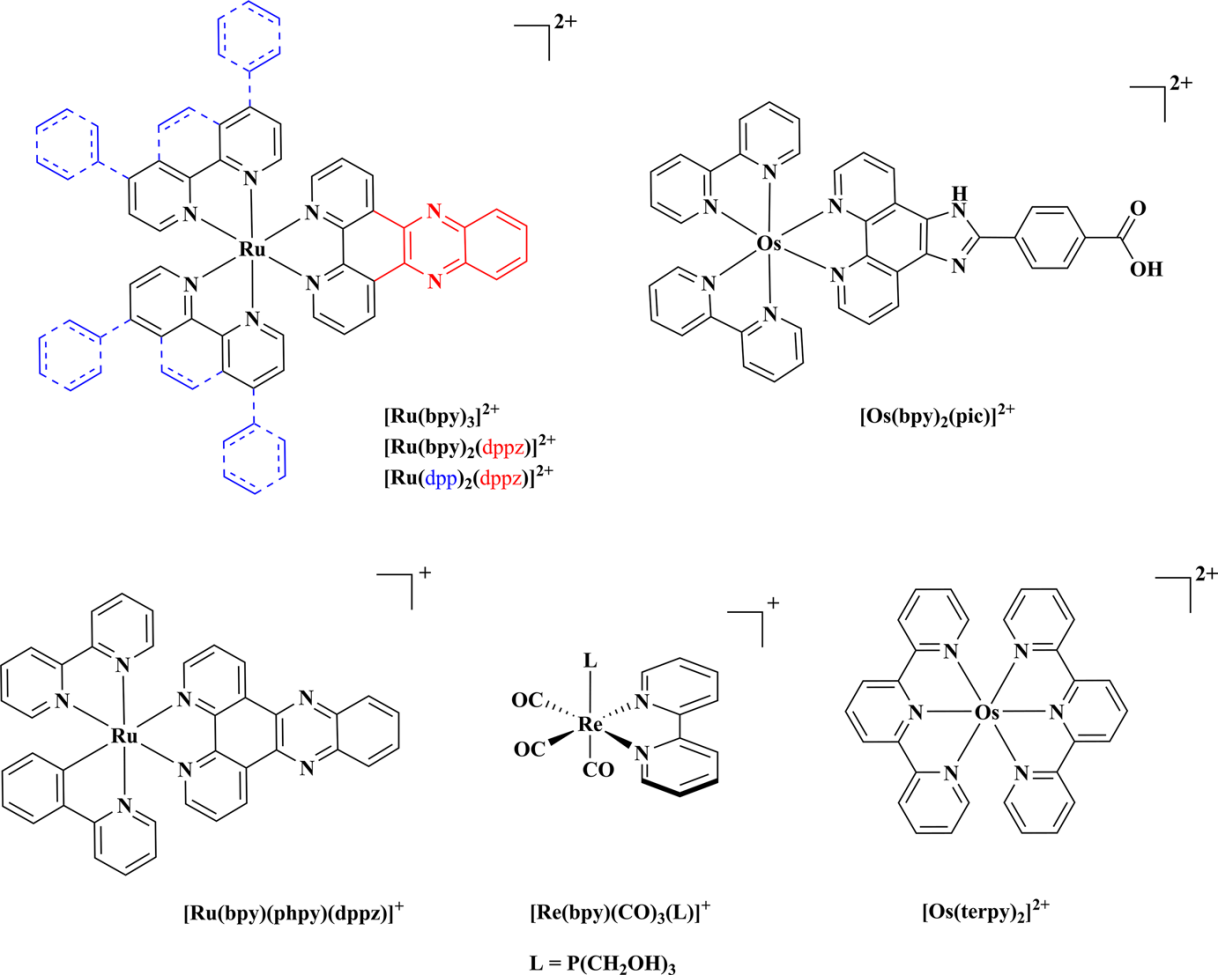
Chemical structures of selected luminescent metal complexes discussed in this section

Figure 2 shows examples of luminescent metal complexes discussed in this section, highlighting key ligands used as building blocks for the design and development of metal complex luminophores.

Aside from their favourable photophysical properties, which are highly tunable due to the synthetic versatility of transition metal luminophores, metal complexes can also show good and also tuneable aqueous solubility, cell permeability and uptake and can be driven to subcellular structures through a range of approaches, in particular, as discussed in this review, by bioconjugation to peptides.

### Tuning of Photophysical Properties

The photophysics and photochemistry of the prototype metal complex [Ru(bpy)_3_]^2+^ has been very thoroughly studied, and it is often used as an example to describe the photophysical activity of Ru(II) complexes [11–14]. The ultraviolet spectrum of [Ru(bpy)_3_]^2+^ is dominated by intense π–π* ligand bands and the broad metal-to-ligand charge transfer (MLCT) transitions in the visible region. Spin-forbidden transitions are facilitated by spin orbit coupling which can be very large for second and third row transition metals such as Ru(II) and Os(II) complexes. Upon photon absorption, the singlet ^1^MLCT excited state is populated and undergoes rapid intersystem crossing (${\text{k}}_{\text{ISC}}$), populating a triplet MLCT (^3^MLCT) excited state with unity quantum yield. In the case of [Ru(bpy)_3_]^2+^, deactivation from the lowest excited MLCT state to the ground state (^1^A_1g_ in O_h_ symmetry) is observed through emission or non-radiative decay via thermally activated (E_a_) population of the ^3^MC state (^3^T_1g_ in O_h_ symmetry). This latter process can lead to ligand dissociation. Indeed, enhanced ligand dissociation following ^3^MC population is observed for sterically strained complexes or for complexes coordinated to ligands with a weak σ donor such as in Ru(II) 2,2′-biquinoline (biq) complexes [15], where reduced ligand field splitting capacity reduces the energy of the dissociative ^3^MC, facilitating its thermal population from the ^3^MLCT state. This is, for example, observed in [Ru(tpy)_2_]^2+^, where tpy is terpyridine, which exhibits a weak short-lived emission at room temperature [16]. The rigid tpy ligands cause geometric distortion from the ideal O_h_ geometry and smaller N–Ru–N trans angles (158.6°) that give rise to a weaker ligand field reducing the energy of the ^3^MC state, thus facilitating radiationless deactivation [17].

Substitution at the 4′ position of the terpyridine ligands in [Ru(tpy)_2_]^2+^ with electron donor or acceptor moieties can enhance the excited state lifetime [18] by destabilising the metal-based highest occupied molecular orbital (HOMO) or stabilising the ligand-based lowest unoccupied molecular orbital (LUMO), respectively. A review by Medlycott and Hanan reports on the various synthetic strategies used to enhance the room-temperature photophysical properties of Ru(II) complexes of tridentate ligands [17].

Emission from Ru(II) complexes typically occurs in the wavelength range of 580–800 nm with λ_exc_ ≈ 400 to 550 nm. Luminescence lifetimes are typically on the order of hundreds of nanoseconds with quantum yields of 1–5% (e.g. [Ru(bpy)_3_]^2+^; *φ*_air_ = 0.04 in water [19]). Molecular brightness, which is defined as the product of molar extinction coefficient (*ε*) and quantum yield (*φ*), is an important photophysical characteristic of an imaging probe, as it can determine the sensitivity and signal-to-noise ratio for luminescence detection. Molar extinction coefficients for Ru(II) complexes are in the range of 5000–20,000 M^−1^ cm^−1^ meaning, their molecular brightness is moderate when compared to organic dyes such as fluorescein. Although less optically tuneable than Ir(III), modification of the σ-donor or π-acceptor properties of Ru and Os complexes can also be used to tune the photophysics of these complexes. For example, coordination of strong π-acceptor ligands, such as 2,2′-biquinoline (biq), decreases ligand field strength and stabilises dπ orbitals, leading to red shifts in absorption and emission of Ru(II) complexes [20]. While, as described above, this can promote population of the ^3^MC state, simultaneous co-coordination of a strong σ-donor ligand such as pyridyl-1,2,4-triazolate (trz) will promote photostability by raising the energy of the ^3^MC, thus preventing both thermal population of this state and potential photodecomposition. Thus, strategic co-mixing of ligands can promote red emission whilst impeding photoinstabilty [20, 21].

Alternative strategies to obtain Ru(II) complexes with NIR emission have included widening the bite angle of the coordinated ligand to increase the ligand field. For example, as mentioned, [Ru(tpy)_2_]^2+^ complex is short-lived with a weak emission at room temperature [16], whereas [Ru(terpy)(dgpy)]^2+^ (dgpy = 2,6-diguanidylpyridine and terpy = substituted 2,2′:6′,2′-terpyridine) complex exhibits a NIR emission at 900 nm [22]. Widening of the ligand bite angle, such as in the case of [Ru(bqp)_2_]^2+^ (where bqp = 2,6-bis(8′-quinolinyl)pyridine), results in red shifted and long-lived luminescence at room temperature (*τ* ≈ 3 μs) [23].

While molecular brightness is modest for most Ru(II) complexes, this is less of an issue for Ir(III) complexes, and they are also inherently more sensitive to the impact of ligand modification due to mixing of ligand and metal states. Factors such as absorbance and emission maxima and molecular brightness are relatively easily modified through ligand modification. The excited states of Ir(III) complexes frequently contain mixed contributions from both ^3^LC and ^3^MLCT and permit greater photophysical tuning, leading to complexes with a diverse range of emission properties across the visible to NIR spectrum. The photophysical properties of such complexes can be tuned via a number of strategies via π-extension of the coordinated ligands, or their modification with electron donating/withdrawing substituents to cyclometalated ligands or by introducing an ancillary ligand, e.g. N, N coordinating ligands that have σ-donating or п-accepting properties [24–27]. A recent review on NIR-emitting Ir(III) complexes discusses in detail the different methods that can be utilised to tune the photophysics of Ir(III) complexes [28]. Of note, although Ru(II) complexes are typically weaker emitters and less amenable to photophysical tuning than Ir(III) complexes, an advantage is that they tend to exhibit lower cytotoxicity upon uptake into cells [29, 30].

Shifting the emission maxima toward the NIR region can also be achieved by selecting an alternative metal centre. Os(II) polypyridyl complexes exhibit emission typically centred in the NIR region (> 730 nm), which is advantageous in the context of bioimaging, including cellular and tissue imaging [31–35]. Os(II) complexes share many of the same photophysical properties with their ruthenium analogues, with some key differences; The ^3^MC state is higher in energy in Os(II) complexes due to increased crystal field splitting that raises the energy of the anti-bonding e_g_^*^ levels, making it thermally inaccessible from the emitting ^3^MLCT state. Thus, Os(II) complexes are extremely photostable, and their photophysics tend to show weak temperature dependence compared to their ruthenium analogues [16].

However, in comparison to [Ru(bpy)_3_]^2+^, the ^3^MLCT excited state lifetime of Os(II) is much shorter-lived, and quantum yields are lower. This is a feature of the energy gap law which comes into play for red to NIR emission. It predicts that the non-radiative rate decay increases as the energy gap between the excited and ground state decreases [36]. Therefore, the low-energy MLCT in the case of Os complexes leads to efficient non-radiative decay.

The bis-terpyridine [Os(tpy)_2_]^2+^ complex, in contrast to [Ru(tpy)_2_]^2+^, exhibits an intense long-lived luminescence at room temperature due to the greater ^3^MC/^3^MLCT energy gap [16].

Amongst the d^6^ complexes, rhenium(I) complexes, *typically rhenium fac tricarbonyl polypyridyls*, also exhibit attractive photophysical properties, including large Stokes shifts, long-lived oxygen sensitive emission, and high photostability. Thus, they have also been applied as bioimaging agents. Photophysical tuning of rhenium complexes is more challenging compared to complexes of Ru(II), Ir(III) and Os(II). In particular, the absorption of such complexes tends to be toward the UV or blue spectral range which limits suitability for imaging applications, especially in tissues. Nonetheless, NIR emission can be achieved by implementing the complex into a D-π-A system [37]. Due to the isostructural relationship between rhenium and technetium-99 m and their characteristic infrared absorption bands, complexes of rhenium(I) have been applied as probes for radio imaging and vibrational imaging, respectively [38, 39]. Furthermore, rhenium(I) tricarbonyl complexes have been developed as agents for photodynamic therapy as they tend to be strong photosensitisers for singlet oxygen generation [40, 41].

Pt(II)/Pt(IV) compounds have historically found application, mainly in therapy as anticancer agents [42, 43], but have also been studied more recently in the context of imaging [44–46]. Luminescence and biocompatibility are prerequisites for use in imaging, and numerous kinetically stable and emissive Pt(II) complexes have been reported. Pt(II) (d^8^) luminophores are distinctive from the complexes discussed above because of their square planar geometries, and Pt(II) luminophores have been based mainly on the general structures [Pt(C^N^N)(L)]^n^ (C^N^N = aryl-substituted N^N ligand, L = monodentate ligand and n = 0 or + 1), cyclometalated tridentate (e.g. [Pt(C^N^C)(Cl)]) derivatives [47]. Tetradentate ligand and π-conjugated porphyrin coordinated Pt(II) luminophores have also been reported [48, 49]. Emission from cyclometalated tridentate complexes is usually attributed to a triplet intra-ligand charge transfer excited state (^3^ILCT), and so photophysical properties are tuneable through ligand modification [44]. π-Conjugated Pt(II) porphyrin complexes, in particular, can exhibit high quantum yields and NIR emission, but efficient and uniform cellular uptake can be problematic due to the large size of porphyrins. In addition, complexes of platinum(II) exhibit a square planar coordination geometry that can permit self-assembly by non-covalent π–π and/or Pt(II)–Pt(II) interactions and the prospect of triplet metal–metal-to-ligand charge transfer (^3^MMLCT) excited state emission [50, 51].

Complexes of Rh(III) [52] and Zn(II) [53, 54] have also been applied in bioimaging, but to date, to a lesser extent than the above metals.

### Reducing Toxicity by Ligand Modification

It is important to consider potential cytotoxicity, both dark and photo-induced, when designing a metal complex luminophore for bioimaging and sensing. The metal centre and coordinated ligands dictate the excited state and redox properties of a complex, and these features, along with size, lipophilicity and overall charge, can generally influence cytotoxicity.

Owing to their long-lived triplet excited states, Ir(III), Re(I) and Ru(II) complexes can induce cellular toxicity via a number of photochemical and photophysical routes. For example, incorporation of tap or hat ligands (tap = 1,4,5,8-tetraazaphenanthrene, hat = 1,4, 5, 8, 9,12-hexaazatriphenylene) in complexes of Ru(II), permits efficient proton-coupled electron transfer (PCET) reactions with bio-relevant molecules such as DNA which can lead to cytotoxic effects. For example, the complexes [(N^N)_2_Ru(tatpp)]^2+^ (where N^N = bpy or phen and tatpp = 9,11 ,20,22 -tetraazatetrapyrido[3,2-a:2′,3′-c:3′′,2′′-1:2′′′,3′′′-n]-pentacene) were shown to cleave DNA through a redox-mediated mechanism [55]. Sensitisation of reactive oxygen species (ROS), such as singlet oxygen, is another important route to photo-induced toxicity that can be exploited in photodynamic therapy applications [56, 57]

Another strategy to phototherapy and a route to cytotoxicity is photo-induced ligand dissociation or substitution which, as previously mentioned, is observed mainly in Ru(II) complexes upon thermal population of the ^3^MC states [15, 58]. Turro et al. investigated in detail the factors that affect ligand dissociation and singlet oxygen generation and demonstrated how ligand tuning can be used to promote both reactions. As the ligand dissociation is thermally driven, it can be quite prevalent under cellular imaging conditions and used for application as such complexes as prodrugs [15, 59–62]. For example, Glazer et. al. have described a series of sterically strained Ru(II) complexes that exhibit dramatically increased ligand photo-release which results in a reactive ruthenium-aquo complex that can photo-bind DNA/biomolecules and trigger cellular apoptosis [63]. Conversely, osmium luminophores tend to be very photostable and relatively inert towards ligand substitution, thus reducing cytotoxic effects that occur through ligand dissociation routes [64, 65].

Lipophilicity often determines cellular uptake and influences intracellular accumulation and localisation, thus affecting toxicity. Coordination to cyclometalated ligands has been shown to enhance lipophilicity and improve cellular uptake for iridium and ruthenium complexes [66, 67]. For example, exchanging the bpy ligand in [Ru(bpy)_2_(dppz)]^2+^ for the cyclometalating 2-phenylpyridine (phpy) ligand yielded a lipophilic and cell-permeable Ru(II) complex [68]. However, the enhanced lipophilicity with cyclometalation can promote toxicity [69, 70].

While the nature of the ancillary ligands is important, chemical modifications to the ligand itself can also influence the lipophilicity and consequently cellular uptake, localisation and cytotoxicity of the complex [71–73]. Increased lipophilicity and dark cytotoxicity was observed for Ru(II) bis-phen and bis-TAP complexes coordinated to a hydrophobic alkylamide phen ligand [74].

Glazer et al. reported on the uptake of two Ru(II) complexes differing in their charge but coordinated to the highly lipophilic *dip* (or *dpp*)* ligand* [75]. The complexes were successfully internalised by A549 cells where the lipophilic [Ru(dip)_3_]^2+^ (log *P* =  + 1.8) accumulated at the mitochondria and lysosomes, while the anionic and less lipophilic [Ru((SO_3_)_2_-dip)_3_]^4−^ (log *P* = −2.2) localised in the cytosol and was mitochondrial-excluding. Both complexes showed photo-induced toxicity, but interestingly, the mitochondrial accumulating complex also showed dark toxicity with an IC_50_ between 0.62 and 3.75 μM. This study highlights the importance of balancing charge and lipophilicity in order to modulate accumulation and limit cytotoxicity (Table 1).

**Table 1:** Lipophilicity and cytotoxicity of selected metal complexes upon synthetic modifications

Compound	log *P*_*o/w*_^a^	IC_50_ (μM) [cell line]	References
[Ru(**phen**)_3_]^2+^	−0.33	268.0 [MCF-7]^b^	[76]
[Ru(**bpy**)_3_]^2+^	−0.41	341.5 [MCF-7]^b^	[76]
[Ru(**pic**)_3_]^2+^	+2.67	66.0 [MCF-7]^b^	[76]
[Ru(**bpy**)_2_(dppz)]^2+^	−2.50	159.9 [HeLa]	[68]
[Ru(**dip**)_2_(dppz)]^2+^	+1.30	–	[77]
[Ru(**dip**)_2_(dppz-**NH**_**2**_]^2+^	−0.27	> 100 [HeLa]^b^	[73]
[Ru(**dip**)_2_(dppz-**CH**_**2**_**OH**)]^2+^	−0.62	Cell-impermeable [HeLa]	[73]
[Ru(bpy)(**phpy**)(dppz)]^1+^ phpy = 2-phenylpyridine	+1.00	0.6 [HeLa]	[68]
[**Os**(**phen**)_2_(phpy)]^+^	+2.43	0.4 [A172]^b^	[78]
[**Ir**(**phen**)(C^N)_2_]^+^ *where N^C* = *2-(p-tolyl)pyridine*	+0.63	1.68 [HeLa]^c^	[79]

^a^Lipophilicity, log *P*_o/w_, was estimated by the partition coefficient of each compound in octanol/water. Propidium iodide and Hoechst are both commercially available organic nucleic acid markers where the first is permeant only to damaged/dead cells, and the latter is cell-permeable. IC_50_ values for the metal complexes were determined based on incubation periods of 24 h unless stated otherwise where ^b^48 h, ^c^72 h

Recently, Finn et al. reported on functionalised Ru(II) complexes with pendant and lipophilic alkyl-acetylthio chains of varying lengths [80]. The complexes were capable of self-assembling into micelles under aqueous conditions and could traverse the cell membrane.

Polyethylene glycol has been conjugated to metal complexes to increase aqueous solubility and reduce dark cytotoxicity [81]. Reduced cytotoxicity was observed for cell-permeable Ir(III)-poly(ethylene glycol) (PEG) conjugates in comparison to the PEG-free counterparts [82, 83]. The long PEG chains likely protect the Ir(III) complexes from non-specific interactions with proteins, DNA and membranes within the cell.

## Rationale for Peptide Conjugation to Transition Row Metal Complexes

Cell membrane permeability is a key barrier to the widespread application of metal complexes in cellular and tissue imaging. One widely used approach to overcome this challenge is the use of organic solvents such as dimethyl sulfoxide (DMSO) or detergents such as Triton-X to permeabilise the cell membrane of mammalian cells. Permeabilising agents act by disrupting the integrity of the membrane bilayer, thus promoting entry of the compound into the cell [84]. This approach is widely used for both organic fluorophores [85–87] and metal complexes [88–91], though it is not always explicitly explained. A key drawback is that above relatively low volume percentages, e.g. for DMSO > 5% vol/vol, solvent permeabilisation can cause irreversible damage to the cell membrane [92], so the approach should be used with care in the study of cultured cells and organic solvent as a permeant is of limited use in tissue or in vivo applications [93, 94].

Other approaches to improving permeability have focused on tuning the lipophilicity, charge and solubility of the complex which in turn can influence cellular uptake and accumulation, as mentioned previously. The use of nanocarriers [95–98], liposomes [99], dendrimers [100], sugars/carbohydrates [101, 102], polyethyleneglycol (PEG) chains [81, 82], vitamins [103–105], antibodies [106], lipophilic moieties such as triphenylphosphonium (TPP) [107], amino acids [108] and cell-penetrating peptides (CPPs) [81, 109–111] has also been shown to increase solubility and improve membrane permeability, facilitating reliable uptake of complexes within cells for a range of applications. Recent reviews describe the preparation and application of ruthenium bioconjugates [112] and vectorisation strategies of metal complex luminophores [3].

Following cellular uptake, subcellular targeting of organelles, such as to the mitochondria or nucleus, is typically of interest in the context of bioimaging/sensing and therapy.

The nuclear envelope is a double membrane comprising of inner and outer nuclear membranes that converge at several sites, generating nuclear pores [113]. Uptake of ions and small molecules is mediated through the nuclear pores through a channel (~ 30 nm in diameter) via passive diffusion. In contrast, uptake of larger molecules is mediated through transport receptors [114]. The mitochondria also feature a double-membrane boundary, though structurally different to the nucleus. The inner mitochondrial membrane is far less permeable than the outer, allowing only very small molecules to cross into the matrix where mitochondrial DNA (mtDNA) and other molecules of analytical interest are contained.

Peptide conjugation has emerged in recent years as a key enabling tool to promote cell uptake, particularly of non-membrane-permeable metal complexes or to enhance uptake and targeting of permeable complexes [115]. The mechanism by which peptides facilitate transport across the cell membrane is often linked to an energy-dependent process such as endocytosis. For example, the recognition of molecules by specific receptors located on the surface of the cell membrane can lead to a receptor-mediated endocytic pathway of uptake. In principle, it is possible that the peptide may lower the uptake efficiency in comparison to the peptide-free complexes—for example, in instances where the complex is highly permeable through the membrane by passive diffusion due to their lipophilic character or in comparison to membrane modification methods such as use of permeabilisation agents. Lower uptake efficiency, however, is often balanced by improved precision in intracellular localisation and decreased cytotoxicity.

Peptide conjugation to metal complexes has been facilitated by a plethora of peptide coupling reactions available to couple peptides to metal complexes, including, but not limited to, amine/carboxyl coupling reactions, “click” chemistry and Sonagashira coupling reactions.

Cell-penetrating and signal peptides specifically are proven reliable vectors for the efficient intracellular delivery of different metal complexes and for targeting organelles with complex membrane structures such as the mitochondria or the nucleus [10, 109, 116–119].

### Peptides

Peptides, short sequences (< 50) of amino acids linked by amide bonds, are physiologically important biomolecules that serve in signalling processes and are ligands for many proteins. In the body, they function as hormones, inhibitors, antibiotics and anti-inflammatories, and both natural and synthetic peptides are finding increasing use in therapeutic applications [120]. Peptides have been widely applied in the pharmaceutical industry to promote permeation of drugs across the membrane; in particular, cell-penetrating peptides (CPPs) have been very effective in this regard and have been applied both as appendages to therapeutic molecules and incorporated into nanocarriers [121]. One of the reasons that peptides have become so important in pharmaceuticals as cargo carriers is that they can be readily accessed, including linear and cyclic and branched peptides, through chemical synthesis. The most important route is through solid-phase peptide synthesis (SPPS), and for many peptide sequences, the synthesis can be automated with continuous improvements reported to protocols that lead to gains in speed, purity and yield. Furthermore, functional terminal groups can be readily appended in the synthesis protocol to facilitate conjugation [122]. The success of peptides in the pharmaceutical industry and also their application in driving organic imaging agents into cells has led to their application in recent years as conjugates to metal complexes to promote their cellular access and targeting.

#### Cell-Penetrating Peptides (CPPs)

The ability of cationic peptide sequences to cross the cell membrane and facilitate uptake of small molecules was first demonstrated in 1965 by Ryser and Hancock with the cationic amino acid-mediated enhanced uptake of albumin followed by studies on conjugation of poly-l-lysine to albumin and horseradish peroxidase [123, 124].

The most studied cell-penetrating peptide is likely the arginine-rich HIV-Tat transduction protein (*RKKRRQRRR*) from the human immunodeficiency virus [125, 126]. Homopolymers of arginine (polyarginines) have shown superior cellular uptake compared to other cationic analogues such as ornithine and histidine [127]. With studies showing no strict requirement for side chain length or backbone chirality (*D-Arg* vs *L-Arg*), it was concluded that the guanidinium head groups of arginine units are the structural features crucial to cellular uptake. Barton et al*.* first reported peptide-facilitated cellular uptake of rhodium complexes [52]. In order to reduce nonspecific DNA binding owing to the highly charged R8, a shorter peptide sequence, RrRK (where r = d-arginine), was conjugated to a Ru(dppz) complex, achieving cellular uptake and nuclear accumulation above a threshold concentration of 100 μM in complete media [128]. Cargo transduction occurs for arginine sequences of Arg_n_ or R_n_, where *n* = 6–11 residues, with octaarginine (Arg_8_) and nonaarginine (Arg_9_) being most efficiently transported. Our group has reported the efficient transport of an otherwise cell-impermeable Ru(II) polypyridyl complex, [Ru(bpy)_2_(pic)]^2+^, via conjugation to octaarginine [88]. The conjugate was found to passively transport into myeloma cells within 12 min. In addition, studies showed that Arg_5_ or lower conjugates are not effective in promoting metal complex permeation. Wender et al*.* reported similar decrease in uptake efficiency of shorter polyarginines [129]. Polyarginine sequences have been extensively explored for promoting or enhancing cellular uptake of metal complexes with applications ranging from bioimaging to medicinal chemistry [29, 88, 116, 130–133]. More specifically, octaarginine-driven cellular uptake has been reported for a range of otherwise impermeable luminescent complexes differing in their metal centre (e.g. Ru(II), Os(II), Ir(III)) *and* coordinated ligands [10, 88, 116, 117, 131]. The effect of conjugation to peptides on the DNA recognition properties of Ru(II) and Ir(III) complexes has also been explored [30, 116, 134]. Recent studies showed that appending an R8 tail to the Ru(II)-dppz complex increased its affinity for G-quadruplexes and that both the ancillary ligand and the octaarginine tail were key to control the selectivity between quadruplexes [134].

We have also demonstrated that two tetraarginine sequences across a linear osmium(II) complex promote cellular uptake, whereas the analogue containing R8 at each terminal was membrane-impermeable. Our data indicated that a contiguous structure may not be required for octaarginine-facilitated transport and that there is an upper limit to the arginine chain length effective in promoting membrane transport of the metal complex [10].

There have been multiple pathways and mechanisms proposed to explain polyarginine CPP behaviour. Although there are a number of studies that report that polyarginines can promote permeation through a passive mechanism [135] or through local changes at the membrane [136], the key pathway in live cells appears to be ATP-activated endocytosis [137]. Polyarginine interactions with cell surface lipids and formation of neutral complexes that transport across the bilayer have also been reported, as well as surface attachment through interactions with heparan sulfate proteoglycans (HSPG) [138–142].

**Fig. 3:**
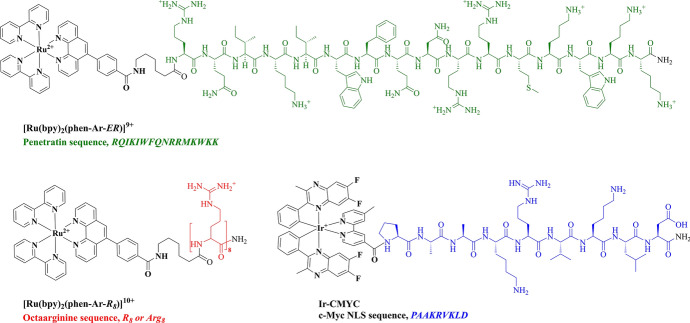
Chemical structures of peptide conjugates [Ru(bpy)_2_phen-Ar-ER)]^9+^ cells [117], Ru(bpy)_2_phen-Ar-R_8_)]^10+^ cells [117] and Ir-CMYC [153]

*Penetratin*, a cationic peptide sequence (*RQIKIWFQNRRMKWKK*) corresponding to the R-helix of the Antennapedia homeodomain, is capable of crossing lipid bilayers and is also quite widely applied cell-penetrating peptide [143]. Studies have shown that the uptake mechanism involves direct interaction of the peptide with membrane lipids and does not involve vesicle disruption or pore formation [144, 145]. Peptide conjugation of penetratin to [Ru(bpy)_2_-phen-Ar-COOH]^2+^ (Fig. 3) allowed for delivery of the complex to the endoplasmic reticulum in live HeLa cells [117].

#### Signal Peptides

Although cell-penetrating peptides such as polyarginines can facilitate efficient cell permeability, more targeted subcellular organelle targeting of imaging probes or theranostic agents can be achieved using signal peptides. Natural signal peptides are amino acid sequences appended to the N termini of newly synthesised proteins in the ribosome that direct the protein from the ribosome along its secretory pathway to its destination. Such signal peptides can provide a powerful means of directing exogenous probes to their target within the cell, and naturally derived peptides have been applied in this regard, and designed sequences have been shown to be recognised by proteins in organelle membranes [146].

Nuclear localisation signal (NLS) peptides derived from transcription factors can cross the cellular membrane and enter the nuclear envelope. To date, NLS sequences that have been derived from transcription factors include NFκB, TCF1-α, TFIIE-β, Oct-6 and SV40 [147–149]. Studies by Ragin et al*.* demonstrated that the NLS peptide, *VQRKRQKLMP*, derived from NFκB, was effective in promoting nuclear penetration [148]. Based on this finding, Keyes’s and co-workers exploited NFκB transcription factor bioconjugation for the efficient and selective nuclear uptake of Ru(II) complexes [2, 119, 150]. Gasser et al. reported on functionalisation of Ru(II) complexes with a nuclear localisation signal peptide (*L*-Arg-*D*-Arg-*L*-Arg-*L*-Lys-CONH_2_) linked via a photolabile protecting group (PLPG); the resulting conjugates showed preferential nuclear accumulation in HeLa and MRC-5 cell lines [151]. A study by the same group described the nuclear delivery of derivatised Re(I) quinolyl complexes using the nuclear localisation sequence, *CRRRK* [152].

In a recent study by Pope et al., an alternative nuclear localisation sequence, *PAAKRVKLD* (Fig. 3), was conjugated to a cyclometalated iridium(III) complex [153]. The c-Myc NLS is derived from the human c-Myc protein and is essential for its nuclear localisation [154]. The Ir-CMYC conjugate was efficiently delivered to the nucleus of human fibroblast cells and was essentially non-toxic, in contrast to the peptide-free parent complex [153].

Cellular uptake of cyclometalated iridium(III) complexes upon conjugation to an endoplasmic reticulum (ER)-targeting sequence (KDEL) and the NLS *PKKKRKV* (derived from SV40 large T antigen) has also been explored [155]. Interestingly, although the ER-targeting conjugate accumulated at the endoplasmic reticulum, the NLS conjugate showed non-specific staining attributed to endosomal trapping upon uptake. Ypsilantis et al. presented a detailed study of the interaction of diruthenium complex peptide conjugates with an oligonucleotide duplex and found that the tethered peptide Gly^1^-Gly^2^-Gly^3^-Lys^1^ CONH_2_ hindered complex binding [156].

Mitochondria-penetrating peptides (MPPs) have been employed for the specific targeting of mitochondria for imaging and therapy. Kelley et al. carried out a detailed iterative study on synthetic peptide sequences relating to signal sequences effective in promoting mitochondrial targeting of fluorescent probes/drug analogues [157]. Amongst the most effective of the sequences studied was an 8-amino acid sequence, *FrFKFrFK*, containing *D*-arginine and hydrophobic residues [157]. Keyes et al. exploited this sequence and the acetyl-blocked sequence, *FrFKFrFK(Ac)*, to effectively and selectively drive mono- and dinuclear Ru(II) complexes to the mitochondria of mammalian cells[118, 119]. As discussed in detail in later sections, such MPP-driven complexes have been applied as bioimaging and sensing tools in live mammalian cells. For example, [(Ru(bpy)_2_phen-Ar)_2_-MPP]^7+^ showed dynamic response to variations in oxygen and ROS levels [118], whereas [Ru(dppz)(bpy)(bpy-Ar-MPP)]^5+^ was used as a light switch probe for mitochondrial nucleoid imaging [119]. Bis-conjugation of the MPP sequence to an achiral Os(II) complex generated a NIR probe showing concentration-dependent cell death that could be tracked on the basis of probe localisation using confocal microscopy, offering a potential theranostic probe [158].

#### Receptor-Targeting Peptides

The peptide sequence Arg-Gly-Asp (RGD) has been applied to mediate specific binding with integrin receptors and has been extensively used in cancer drug research as integrin receptors, such as α_ν_β_3_, which are overexpressed in certain tumour cells [159–161].

Adamson et al. first reported on RGD-labelled luminescent metal polypyridyl complexes [110]. Complexes of ruthenium(II) were conjugated to a linear RGD peptide with the objective of targeting platelet integrin, α_IIb_β_3_ to, through emission anisotropy, reflect integrin conformation status. Integrins are adhesion receptors and transmembrane proteins that undergo large conformational changes and clustering on activation that alters their affinity for their receptors, and RGD is a peptide motif recognised by all integrins [162]. The yielded [Ru(N^N)(pic-RGD)]^2+^ (where N^N = bpy or dpp) conjugates showed high binding affinity and specificity for α_IIb_β_3_, and through alterations in metal complex photophysical behaviour and anisotropy, it was possible to distinguish between different activation states of integrin. A two-step binding was determined for [Ru(dpp)_2_(pic-RGD)]^2+^ with K_d1_ = 0.25 ± 0.29 μM and K_d2_ = 4.37 ± 0.82 μM. Additionally, confocal imaging revealed that both bpy-RGD and dpp-RGD conjugates selectively bind to CHO cells expressing the resting form of α_IIb_β_3_.

In spite of their biomedical importance, there are surprisingly few examples of linear- or cyclic-RGD-metal complex conjugates either applied as therapeutic agents [111, 163, 164], luminescent probes [110] or both [165–167].

A zinc phthalocyanine complex conjugated to a cyclic RGD peptide displayed dramatically higher cellular uptake in α_v_β_3_^+^ U87-MG cells compared with the α_v_β_3_^−^ MCF-7 cells [168]. A recently reported Ru-cRGD (cyclic RGD) conjugate exhibited strong two-photon luminescence and showed preferential accumulation in malignant cells with promising potential as a theranostic agent [167]. In an alternative system, dual-imaging nanoprobes were prepared by conjugating iridium(III), gadolinium(III) and RGD onto silica nanoparticles [169]. The water-soluble particles permitted in vitro and in vivo studies using confocal luminescence imaging and magnetic resonance imaging.

Certain vectors can be used to target cells that overexpress key receptors such as folate, transferrin and somatostatin at the membrane surface of different disease states [170–173]. For example, enhanced uptake of a somatostatin-targeting Ru(II) conjugate was achieved in A549 cells overexpressing somatostatin receptors [173]. Although the conjugate did not act as a bioimaging probe, it showed excellent photosensitised toxicity with an IC_50_ of 300 μM in the absence of light versus an IC_50_ of 13 μM upon irradiation (PI > 23).

Similarly, a redox active Pt(IV) complex was coordinated to the tumour-penetrating sequence (*TKDNNLLGRFELSG*) that targets the membrane protein *heat shock protein 70 positive* (*memHSP70*+) which is upregulated in colorectal cancer cells but is not usually found in healthy tissues [174]. The Pt(IV) complex is reduced in the cell to Pt(II), releasing the axial ligands and leading to cytotoxicity[175].

This strategy of conjugation to tumour recognition or penetrating sequences can also be exploited in the design of targeted probes for bioimaging. For example, C–X–C chemokine receptor 4 (CXCR4) is overexpressed in over 23 different types of cancer and is more prevalent in malignant cancer tissue [176]. With this consideration, a rhenium(I) tricarbonyl complex was conjugated to a derivative of *T140* (14 amino acid sequence), a known antagonist of CXCR4, and showed potential as an imaging agent for CXCR4 expression that was capable of differentiation between cancerous and healthy tissue [177, 178]. Kuil and co-workers had previously presented an iridium(III)–peptide conjugate for FLIM-based visualisation of CXCR4 expression in cells by conjugating the complex to a series of Ac-TZ14011 peptides [179].

Vallaisamy et al. reported on an iridium(III) complex conjugated to the hexapeptide *MKYMVm*, *the peptide agonist*, which selectively targeted formyl peptide receptor 2 (FPR2) in live cells [180]. Formyl peptide receptor plays an important role in chemotactic signals and modulation of host defence and inflammation [181].

**Fig. 4:**
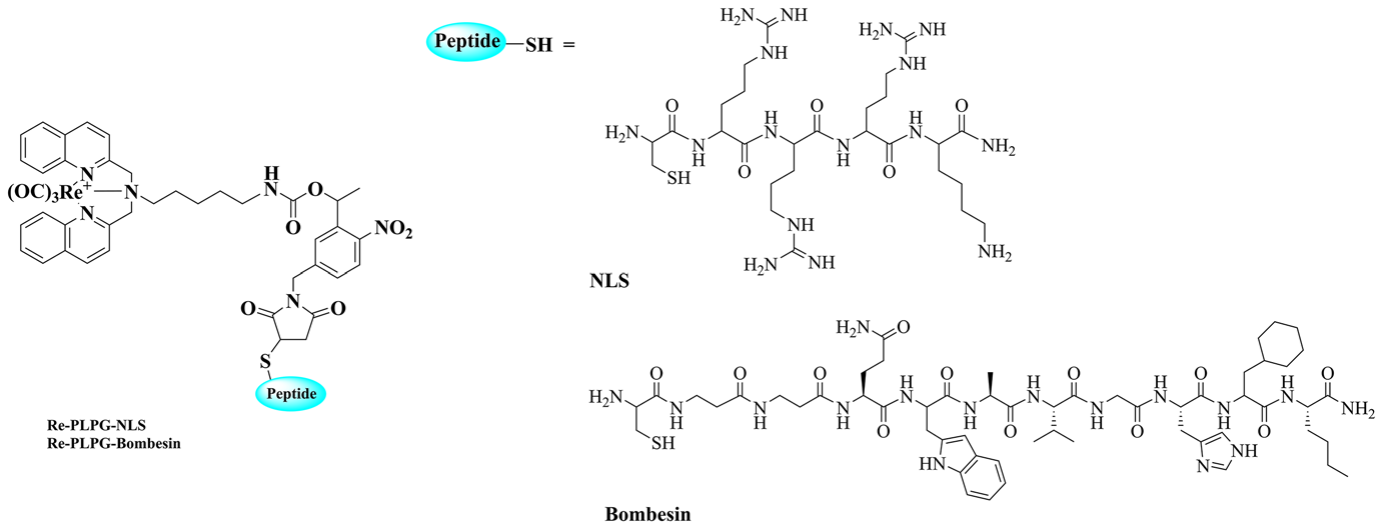
Structure of Re(I) complex conjugated to a nuclear localisation signalling (NLS) peptide or a bombesin (BBN) derivative peptide sequence used to improve uptake of the complex by cancer cells overexpressing the gastrin-releasing peptide receptor (GRPR) [152]

Gasser et al. have functionalised Ru(II) and Re(I) complexes with the peptide bombesin (BBN, 14 amino acid sequence) in order to enhance uptake and accumulation of the complexes selectively in cancerous cell lines [151, 152]. The peptide BBN, Fig. 4, is structurally similar to the human gastrin-releasing peptide (GRP) and is recognised and internalised by GRP receptors that are overexpressed in some cancer cell lines [182, 183].

A series of zinc phthalocyanine peptide conjugates were synthesised to target gastrin-releasing peptide (GRP) and integrin receptors [184] in order to initiate a targeted therapeutic effect.

Agorastos et al. reported a rhenium tricarbonyl complex functionalised with acridine orange that could selectively stain the cell nucleus of both mouse melanoma (B16-F1) and human prostate adenocarcinoma cell line (PC-3) cells [185]. Conjugation of the complex to the bombesin peptide resulted in cell-specific uptake. The peptide conjugate was membrane-impermeable to B16F1 cells, but readily permeated into PC-3 cells. Cell-specific uptake was achieved by exploiting the lack of gastrin-releasing peptide (GRP) receptors in B16F1 cells, but which are expressed in PC-3 cells. Interestingly, conjugation to the peptide prevented the ability of the complex to enter the nucleus.

A short peptide based on the endogenous opioid pentapeptide ligands was chosen for the preparation of luminescent heterobimetallic Ir(III)/Au(I) conjugates that were found to be membrane-permeable, and localise in the lysosomes of A549 cells [186].

A rather different type of recognition occurs in the case of peptide nucleic acids (PNAs). PNAs are non-natural DNA/RNA analogues that consist of *N*-(2-aminoethyl)glycine units which form a pseudopeptide backbone bearing the four nucleobases. They thus exhibit strong affinity for nucleic acid strands [187]. PNA conjugation has been explored in the development of luminescent rhenium-PNA conjugates for cell imaging and DNA targeting [188–191].

Although it is outside of the scope of this review on peptide-driven luminescent metal complexes, it is important to note that there are also several reports on metal polypyridyl complexes conjugated to proteins. For example, Ru(II) complexes have been conjugated to protein G [192], cytochrome c [193] and human serum albumin (HSA) protein [194] for various applications. Chakrabortty et al. presented a protein-Ru(II) hybrid with photosensitising properties which targets the mitochondria [194]. In this case, the Ru(II) complex was conjugated to HSA protein and covalently decorated with mitochondria-directing triphenylphosphine groups, thus achieving cellular uptake and specific subcellular accumulation.

## Luminescent Metal Complex Peptide Conjugates Applied in Bioimaging

Early examples of *peptide-conjugated* metal complexes in confocal imaging are described in studies carried out by Barton et al. Conjugation to octaarginine enhanced cellular uptake of rhodium(III) 5,6-chrysenequinone diimine (chrysi) and ruthenium(II) dipyrido-phenazine (dppz) complexes, and interestingly, attachment of a fluorescein moiety, in the case of the Ru(II)-dppz complex, led to nuclear localisation [52, 130]. Our group has focused extensively on the design and development of peptide metal complex conjugates for bioimaging, sensing and theranostics. A series of otherwise cell-impermeable ruthenium(II), iridium(III) and osmium(II) complexes have been conjugated to cell-penetrating and signal peptides and have been studied using confocal microscopy, lifetime imaging and resonance Raman spectroscopy. Recently, a polyarginine Os(II) probe was used in imaging of pancreatic multicellular tumour spheroids, marking the first step towards the application of such luminescent peptide probes in tissue imaging [10].

### Cytoplasm

**Fig. 5:**
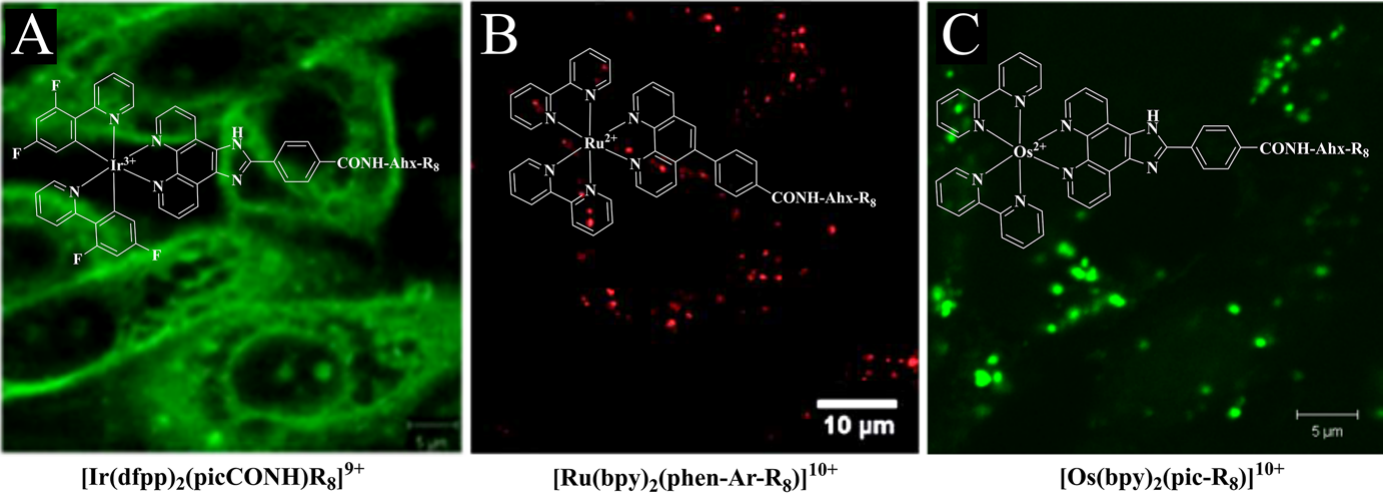
Confocal luminescence imaging of live mammalian cells incubated with octaarginine conjugates of Ir(III), Ru(II) and Os(II). **A** Ir-R8 in CHO cells at 70 μM/15 min incubation [29], **B** Ru-R8 in HeLa cells at 70 μM/4 h incubation (Reproduced from Ref. [117] with permission from the Royal Society of Chemistry) and **C** Os-R8 in CHO cells at 70 μM/2 h incubation in the absence of light at 37 °C (Reproduced from Ref. [131] with permission from the Royal Society of Chemistry)

Octaarginine CPPs drive cellular uptake and internalisation, usually into the cell cytoplasm, resulting in cytoplasmic or non-specific staining. Cell-permeable R8-conjugates have been explored as bioimaging probes, namely [Ir(dfpp)_2_(picCONH)R_8_]^9+^ [29] and Ru(bpy)_2_(phen-Ar-R_8_)]^10+^, for confocal and high-resolution stimulated emission depletion (STED) imaging [117], and [Os(bpy)_2_(pic-R_8_)^10+^ and [Ru(bpy)_2_(pic-R_8_)]^10+^ for confocal and phosphorescence lifetime imaging microscopy (PLIM) studies [131], and [Os-(R_4_)_2_]^10+^ for confocal/lifetime imaging of two-dimensional (2D) and three-dimensional (3D) cell cultures [10]. Figure 5 illustrates the dye distribution of key examples of octaarginine conjugates of Ir(III), Ru(II) and Os(II). Conjugation to Arg_8_ rendered all three complexes membrane-permeable in aqueous solution without the requirement for permeabilisation agent such as detergent or organic solvent. Fei et al. also reported on cytoplasmic and vesicular staining in HeLa cells following incubation with a histidine-targeting Ir(III)-HTat conjugate [195].

A fused peptide consisting of a nona-arginine fragment attached to a sequence (*RHVLPKVQA* = A*β* aggregation inhibitor) with anti-amyloid activity was labelled via a histidine residue to a platinum(II) complex [196]. The resulting luminescent conjugate was studied in cells and was shown to stain the cytoplasm of HeLa cells. In vivo studies in *Drosophila melanogaster* showed that the luminescent platinum conjugate could permeate the blood brain barrier of these organisms and evenly distribute in the brain. This work highlighted the use of fused peptides as vectors to penetrate the blood brain barrier while also selectively targeting biorelevant molecules or, in this case, inhibit the formation of amyloids.

Using “click” chemistry, a rhenium(I) tricarbonyl complex was attached to a lipopeptide known to increase cell permeability [197]. The addition of the myristoylated HIV-1 TAT (myr-Tat) peptide to the rhenium complex substantially enhanced uptake in cells compared to the peptide-free complex and showed cytoplasmic accumulation with partial nucleoli staining.

### Nucleus, DNA/RNA

The interaction of metal complex luminophores with nucleic acid materials has been the subject of extensive study since the 1980s. This has led to deep insight into the nature of metal complex–DNA interactions, expanding the prospects for both intracellular sensing and photo therapy by these species. Increased understanding of the factors that can be used to promote metal complex permeation and organelle targeting have led to the application of such complexes to study nucleic acid materials in cells, with several studies now reporting on the nuclear uptake and staining of metal peptide conjugates used for imaging or sensing of DNA within live cells [198–200].

One of the earliest of such studies was reported by Brunner and Barton who utilised functionalised rhodium complexes with octaarginine peptides to study DNA mismatches [52]. The rhodium complexes were capable of specifically binding to DNA mismatches where they can photocleave the DNA adjacent to the mismatch. The rhodium complex conjugated to an octaarginine peptide appended to fluorescein was rapidly internalised within the cell and localised in the nucleus. It was noted that the presence of the peptide led to binding of matched DNA (electrostatic interaction between peptide and DNA), although the photocleavage only occurred at DNA mismatches as desired.

**Fig. 6:**
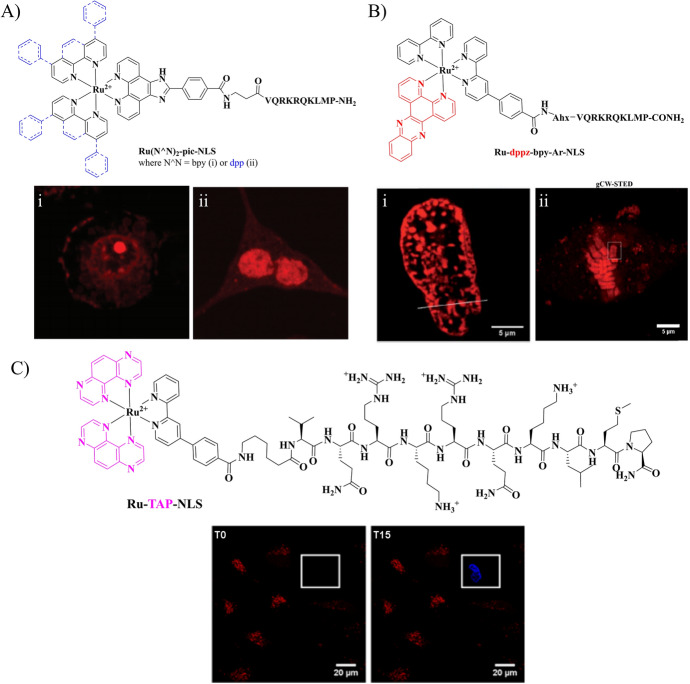
Chemical structures of NLS-driven nuclear targeting Ru(II) complexes and representative images of their corresponding application in mammalian cells. **A**  [Ru(bpy)_2_(pic-NLS)]^6+^(i) and [Ru(dpp)_2_(pic-NLS)]^6+^ (ii) showing nuclei and nucleolus staining, respectively (Reproduced from Ref. [150] with permission from the Royal Society of Chemistry). **B** Confocal (i) and STED (ii) images of [Ru(dppz)(bpy)(bpy-Ar-NLS)]^6+^ nuclear and chromosomal DNA staining (Reproduced from Ref. [117] with permission from the Royal Society of Chemistry). **C** Following DNA binding of Ru-TAP-NLS in live HeLa cells, continuous photoirradiation of selected cells resulted in cellular damage as indicated by DRAQ 7 nuclear staining in blue [2] (Reprinted (adapted) with permission from Burke C. S. et. al. [2]. Copyright 2018 American Chemical Society)

We reported that specific nuclear targeting could be achieved using the aforementioned transcription factor NFκB and that the localisation of the complex in the nucleus seems to be dictated by the lipophilicity of the complex. As shown in Fig. 6, both [Ru(bpy)_2_(pic-NLS)]^6+^ and [Ru(dpp)_2_(pic-NLS)]^6**+**^ (where bpy = 2,2-bipyridine, dpp = 4,7-diphenyl-1,10-phenanthroline and pic = 2-(4-carboxyphenyl)-imidazo-[4,5-f][1,10]-phenanthroline) were efficiently transported across the cell and nuclear membrane in Chinese hamster ovary (CHO) cells [150]. However, the more hydrophilic bpy-NLS showed nuclear staining, whereas the dpp-based conjugate showed accumulation in the nucleolus, thus highlighting that the lipophilic character of the metal complex remains important even in intraorganelle distribution.

High-resolution imaging of chromosomal DNA was achieved using a Ru-dppz NLS conjugate, [Ru(dppz)(bpy)(bpy-Ar-NLS)]^6+^, which also allowed tracking of the different stages of mitosis in HeLa cells using STED [117]. In a separate study, the [Ru(tap)_2_(bpy-Ar-NLS)]^6+^ showed nuclear penetration and DNA binding indicated by the extinguished complex emission [2]. In an example of the multimodal addressability of such complexes, they were confirmed to remain present in the nucleus after emission extinction by resonance Raman microscopy. With the aim of extending the application of Ru-NLS conjugates toward theranostics, it was shown that upon in situ photoirradiation, cellular destruction is accomplished, attributed to DNA oxidation by photo-induced electron transfer from a guanine base and the Ru(II) complex, analogous to a mechanism reported for related tap complexes in solution [201].

A rhenium complex conjugated to an NLS peptide was reported to exhibit nucleolar localisation and efficient singlet oxygen generation under light irradiation in polar (*Φ* = 0.25) or lipophilic (*Φ* = 0.75) environments [202]. This luminescent probe is attractive for dual application in both imaging and photodynamic therapy as it exhibits low dark toxicity (IC_50_ = 35 µM), but enhanced toxicity under UV irradiation. In a separate study, the derivatised and caged Re(I) complex, Re-PLPG, was coupled to an NLS peptide that showed penetration into sub-cellular compartments such as the nucleoli, thus allowing interaction of the complex with nucleic acids [152].

Metal complex peptide nucleic acid (PNA) conjugates are a useful approach to probe different nucleic acid strands due to their ability to hybridise to their complementary oligonucleotide strands with high specificity which is advantageous in sensing and therapy. For example, the Re(I)-PNA conjugate, [(CO)_3_Re(pyridazine-PNA)(Cl)_2_Re(CO)_3_], suitable for two-photon excitation (λ_exc_ 750 nm), revealed cytoplasmic and nuclear staining in HEK-293 cells attributed to PNA–nucleic acid binding [188]. Notably, small concentrations of DMSO were required for uptake of the conjugate. The emission wavelength was substantially altered depending on sub-cellular localisation and could be used to differentiate between the cytoplasm and the nucleus. The difference in emission energy is attributed to the difference in polarity/rigidity between the different locales. A follow-on study by the Licandro research group on related rhenium complexes conjugated to different PNA sequences revealed difficulties in cell studies, including poor solubility and endosomal entrapment [189]. These issues are frequently encountered in biological studies of metal complexes and can hinder sensing and imaging applications due to a low rate of cell uptake and off-target localisation.

### Mitochondria

The nucleus is the primary location of DNA within the cell, but the mitochondria is also an important repository. Although containing much less DNA, it contains 37 genes in total that encode proteins and RNAs critical for energy transduction.

A histidine-binding Ir(III) complex was bis-conjugated to an HTat sequence and a mitochondrial targeting sequence derived from the mitochondrial protein cytochrome P450 [195]. The conjugate was membrane-permeable and efficiently targeted the mitochondria.

**Fig. 7:**
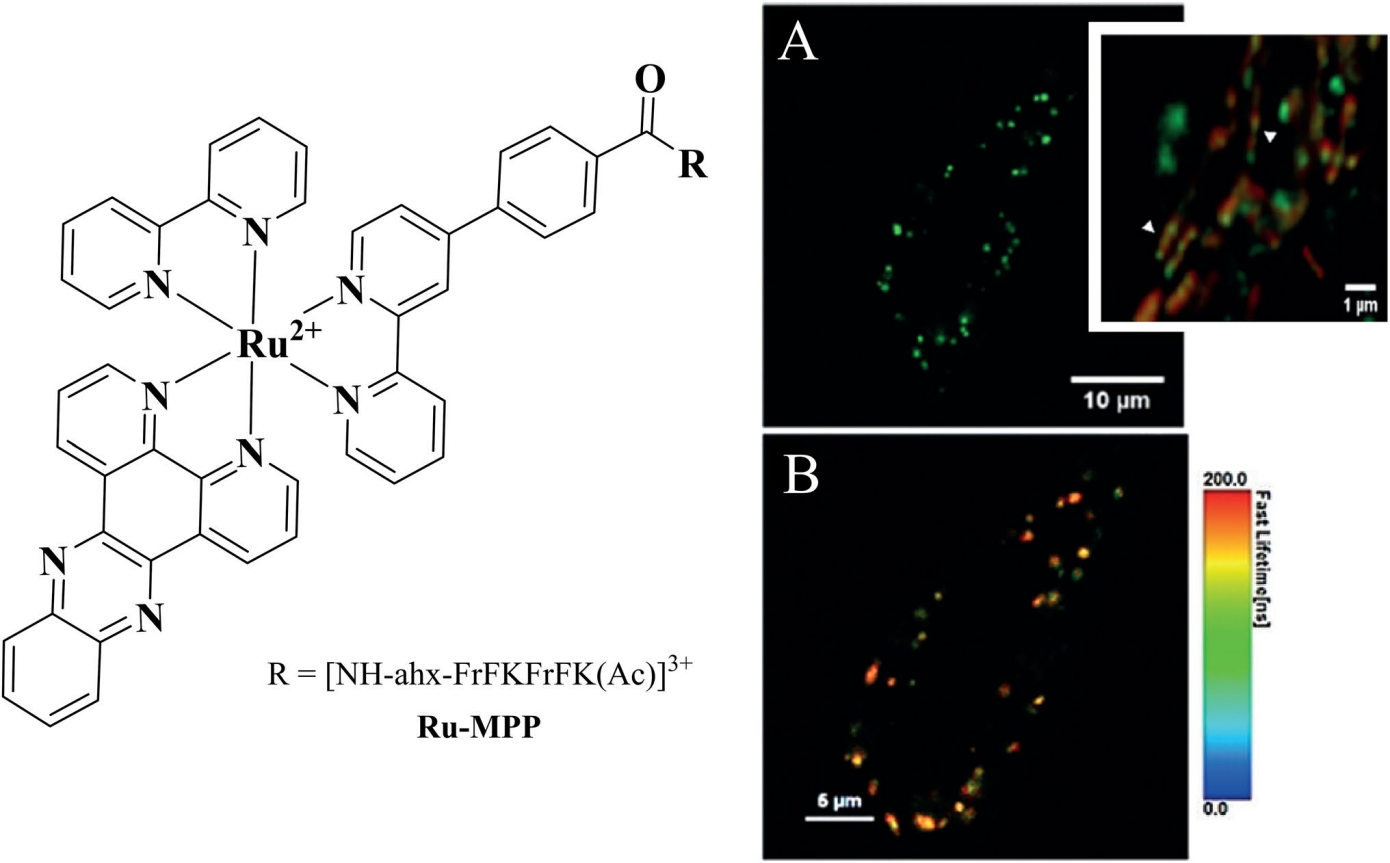
Precision-targeted [Ru(dppz)(bpy)(bpy-Ar-MPP)]^5+^ (10 μM/2 h) light-switching probe in mitochondria of live HeLa cells. **A** Confocal image with inset showing a close-up of the mitochondria co-stained with MitoTracker Deep Red (red) and **B** luminescence lifetime distribution of the conjugate [119]. Reproduced with permission from Angewandte Chemie International Edition

In a recent publication, precision targeting of mitochondrial DNA in live HeLa cells was achieved using an MPP-driven light-switching Ru^II^-dppz complex [119]. Confocal laser scanning microscopy showed rapid cellular uptake of [Ru(dppz)(bpy)(bpy-Ar-MPP)]^5+^ in live HeLa cells, and localisation to mitochondrial sub-structures was confirmed using luminescence lifetime imaging (Fig. 7). Solution titration with ctDNA showed that the DNA binding ability of the parent complex, mediated by dppz intercalation, is retained for the Ru^II^-dppz MPP conjugate. Additionally, an increased binding constant was reported, which was attributed to electrostatic interactions between the polycationic sequence of MPP and the anionic DNA backbone. The conjugate showed low cytotoxicity in the dark and under imaging conditions, thus facilitating mtDNA visualisation. Photo-induced toxicity was observed only under continuous and intense irradiation, enabling controllable initiation of cell death, making it an interesting prospect for theranostic applications.

Recently, the successful conjugation of an osmium(II) complex to two mitochondrial-penetrating peptides was reported [158]. The bis-MPP conjugate strongly confined to the mitochondria at and below concentrations of 30 μM and leached out of the organelles and into the cytoplasm over time. At increased concentrations, it showed cytoplasmic and even nucleoli staining, leading to cell death. This localisation switch was also reflected by the cell death mechanism, where at 30 μM, loss of the membrane potential was observed, whereas at increased probe concentrations, a moderate effect on depolarisation and a greater caspase activity was observed instead.

### Endoplasmic Reticulum (ER)

The endoplasmic reticulum (ER) in eukaryotic cells is the site of synthesis and processing of many transmembrane and secretory proteins, synthesis of lipids and calcium regulation. Accumulation of unfolded or misfolded proteins trigger an ER stress response which regulates cell functions to either restore ER homeostasis or to induce apoptosis for damaged cells. Complexes that target the ER may be used as imaging tools to study the endoplasmic reticulum and processes, such as ER stress, or as therapeutic tools, as the ER signalling pathways have been linked to various diseases, including cancer.

**Fig. 8:**
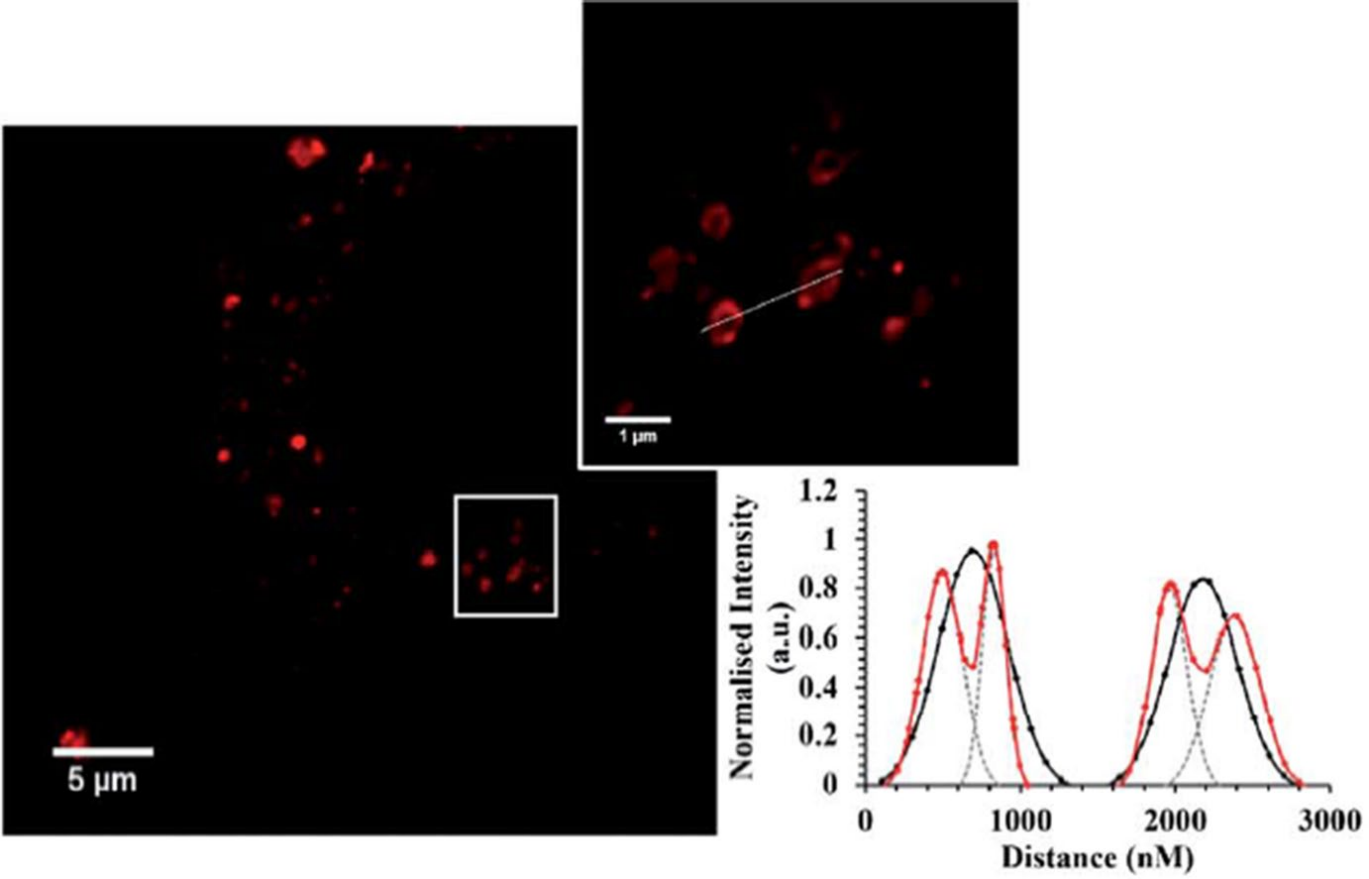
Confocal (left) and STED (inset) imaging of [Ru(bpy)_2_-phen-Ar-ER]^9+ −^ (70 μM/4 h)-stained ER in a single fixed HeLa cell. The plot profile compares the full width at half maximum (FWHM) of confocal (*black coloured em dash*) versus STED (*red coloured em dash*) resolution. Reproduced from Ref. [117] with permission from the Royal Society of Chemistry

The ruthenium(II) complex [Ru(bpy)_2_-phen-Ar-COOH]^2+^, exhibiting an emission maximum at 604 nm, was conjugated to the penetratin/ER cell-penetrating peptide, yielding the [Ru(bpy)_2_-phen-Ar-ER]^9+^ bioconjugate [117]. The conjugate was used for super-resolution (STED) imaging of the endoplasmic reticulum in HeLa cells as shown in Fig. 8. Bright punctuate spots are observed in the confocal image, whereas STED imaging reveals the tubular structures of the ER.

### Lysosome

Lysosomes are subcellular organelles that are surrounded by a single membrane and are characterised by an acidic interior environment (pH ∼ 4.5 to 5), in contrast to mitochondria, for example, which are alkaline (pH ∼ 8). Lysosomes play an important role in cellular processes, including homeostasis [203], energy metabolism [204], enzymatic activity [205] and autophagy [206] which is also related but not limited to inflammatory diseases. Monitoring changes in the lysosomal environment including pH variations can aid understanding of lysosomal function and dysfunction. Furthermore, lysosomes are emerging as attractive therapeutic targets [203]. For example, cyclometalated iridium(III) complexes were applied as pH-activatable cell imaging agents and photosensitisers, highlighting the dual application of such probes for photodynamic therapy and real-time therapeutic monitoring [207].

With the aim of developing potential theranostic agents, Fernández-Moreira et al. reported the preparation of peptide-linked bimetallic Ir(III)/Au(I) conjugates [186]. The luminescent properties of the iridium moiety permitted confocal imaging and tracking of the conjugates upon cellular uptake and lysosomal accumulation, and the coordination sphere of the gold appeared to influence cytotoxic activity. The cysteine-containing conjugate showed antiproliferative activity which is thought to be attributed to the readily cleaved Au–S(cysteine) bond.

A Ru(II)-cyclodextrin-RGD nanoassembly reported by Mao et al*.* was found to accumulate in lysosomes of integrin-rich tumour cells and trigger apoptosis through lysosomal damage, ROS elevation and caspase activation [166]. Uptake of nanoparticles via an endocytic mechanism frequently results in lysosomal accumulation. Endocytosis is generally associated with endosomal entrapment in early or late endosomes which then fuse with lysosomes at a later stage if they are not released to the cytoplasm.

Recently, the bridged octaarginine conjugate, [Os-(R_4_)_2_]^10+^ was found to be taken up initially into the cytoplasm of A549 cells prior to accumulating in lysosomal structures at 30 μM/48 h [10]. This permitted both confocal imaging and luminescence lifetime mapping of the intracellular environment, including potential response to redox species as discussed later. Although lysosomal accumulation is desired in the context of therapy or redox and pH sensing, endosomal entrapment can hinder delivery of a luminophore probe or nanoparticle to the desired intracellular destination. The recently reported RuBDP nanoparticles were found to localise in late endosomes and lysosomes of A549 cells [208]. The particles ratiometrically responded to fluctuations in oxygen concentration and, interestingly, exhibited emission enhancement within 4 h following initial uptake, the origin of which is thought to reflect endosomal escape. Endosomal escape is a topic that is particularly important in drug delivery, and approaches addressing this challenge have focused on promoting endosomal membrane fusion and destabilisation or pore formation in the endosomal membrane [209, 210]. In addition, several endosomal escape agents have been identified, such as chemical agents or viral- and bacterial-derived proteins and peptides [211]. Following protocols emerging in this domain, e.g. through modifying the particle composition to achieve pH-induced release [211, 212], efficient endosomal escape and specific organelle targeting may further expand the application of nanoparticles and probes.

## Sensing Capabilities of Peptide Metal Complex Conjugates

Taking advantage of the excellent targeting capability of peptides, there are several examples of emissive metal complex conjugates that have been applied for sensing of bio-relevant species such as oxygen and molecules including DNA and proteins. The characteristic luminescence lifetime- or intensity-based response typically reflects the interaction of the metal complex with these species within a cellular environment.

Coordination of responsive ligands allows for the design and preparation of complexes with a responsive luminescence. For example, complexes of dipyrido[3,2-a:2′,3′-c]phenazine (dppz) and its derivatives exhibit no luminescence in aqueous solution, but emission is switched on in hydrophobic environments, such as upon DNA binding, leading to the design and development of a range of DNA “light-switch” dppz complexes [213, 214]. Sensing of important molecular structures can be enhanced via coordination of the lipophilic diphenyl phenanthroline (dpp) ligand, for example, which also allows for cellular uptake and targeting of lipid-rich regions [75, 110, 150]. Incorporation of a targeting vector allows for *targeted* sensing. For example, conjugation of a Ru(II) sensor to a mitochondrial-penetrating peptide enables monitoring of *local* oxygen fluctuations in live cells using either emission intensity or lifetime imaging.

### Oxygen

There are several methods applied traditionally for monitoring and measuring dissolved oxygen in biological systems, for example, Clark-type O_2_ electrodes [215], electron paramagnetic resonance (EPR) probes [216] and microelectrodes or needle probes [216–220]. However, there is a demand for less invasive techniques for O_2_ sensing and particularly for sensing modalities that can be readily followed dynamically intracellularly with as little as possible interference with the cell. For this reason, *quenched phosphorescence*-based O_2_ sensors are particularly attractive for intracellular oxygen (icO_2_) sensing. Ideal characteristics of an intracellular oxygen sensor include high oxygen responsivity, photostability, cell uptake efficacy, molecular brightness, biocompatibility, cytotoxicity and subcellular targeting ability where desired.

There are numerous examples of emissive probes that have been applied for *oxygen* sensing using lifetime- or intensity-based methods. As mentioned, a key advantage of lifetime sensing is that emission lifetime is largely independent of probe concentration. A drawback though is that phosphorescence lifetime imaging/sensing requires a microscope coupled with a lifetime/FLIM unit, which is rather a specialist technique, not a routine tool in many bio-laboratories. Whereas, intensity-based sensing can be performed using conventional instrumentation such as a fluorescence microscope or plate reader. Intensity-based sensing measurements, as described, can be applied where the probe species is combined with a reference, which overcomes issues of concentration and other artefacts in an intensity-based measurement. The choice of modality for O_2_ sensing will also depend on the sample for analysis. For example, using a conventional plate reader, intensity-based measurements permit parallel analysis of monolayer cells exposed to various conditions in a single experiment while also carrying out the measurement in multiplicate. For three-dimensional cell models or tissue samples, PLIM is typically the method of choice as it allows visualisation of spatial O_2_ distribution throughout the sample.

Complexes of ruthenium(II), iridium(III) and Pt(II) or Pd(II) porphyrins are amongst the most widely studied oxygen sensors owing to their long-lived triplet excited states which are highly susceptible to oxygen quenching, giving a characteristic lifetime- and intensity- based response to oxygen concentration, as described by the Stern–Volmer equation.

Phosphorescent Pt(II) and Pd(II) porphyrins exhibit phosphorescence lifetimes ranging from 40 to 100 μs and 400 to 1000 μs, respectively [221]. Papkovsky and co-workers have worked extensively on Pt(II) and Pd(II) porphyrin probes which show good photostability and efficient quenching by oxygen [222–225]. The solubility of such probes in water and targeting ability can be improved by conjugation to protein cargos, PEG chains or cell-penetrating peptides [224, 226–229]. Dmitriev et al. presented a Pt(II) coproporphyrin conjugated to a peptide fragment derived from the antimicrobial bactenecin 7 peptide [227]. The conjugate showed efficient cellular uptake across several cell lines, cytoplasmic and mitochondrial accumulation and was used for monitoring intracellular O_2_ levels upon exposure to metabolic stimuli reagents. The application of this conjugate and similar Pt(II) coproporphyrins in cells can be hindered by poor photostability and potential photocytotoxic effects.

Although somewhat limited by their photostability, iridium(III) complexes have shown promise for in vitro and in vivo oxygen and hypoxia mapping and low cytotoxicity against 2D cell monolayers [230–235].

Ir(III) dyads such as the iridium–coumarin ratiometric probe, C343-Pro_4_-BTP reported by Yoshihara et al., have been developed for ratiometric intensity-based O_2_ sensing [236]. In this report, the coumarin moiety (C343) is linked to the iridium (BTP) complex through a tetraproline amino acid linker, and upon excitation at 405 nm, energy transfer from C343 to BTP yields emission from both dyad components at 480 nm and > 610 nm, respectively. The phosphorescence emission signal of the iridium is quenched by oxygen, and the ratio of the emission from the dyad moieties exhibits an O_2_-dependent response both in solution and in live HeLa cells. In later studies, octaproline [234] and octa- and dodecaarginine [237] linkers were utilised in coumarin–iridium(III) dyads in order to enhance cellular uptake to enable ratiometric imaging of the oxygen gradient in HeLa cells.

Several complexes of ruthenium(II) have demonstrated *in cellulo* oxygen response as molecular probes or part of a dual emissive dyad [238–240]. In the context of peptide conjugates, Keyes’ group presented the octaarginine conjugate, [Ru(bpy)_2_(pic-R_8_)]^10+^ [1, 88], whose luminescence lifetime, *similar to the parent complex*, was oxygen-sensitive. Confocal imaging revealed rapid uptake of [Ru(bpy)_2_(pic-R_8_)]^10+^ in myeloma cells and human blood platelets, and lifetime imaging was used for cellular oxygen mapping where, for example, the probe lifetime was shortest (~ 400 ns) when the conjugate localised in the cell membrane. This agrees with the increased solubility of oxygen in the cellular membrane. Although the emission lifetime of this complex is strongly oxygen-dependent, the quenching constant by O_2_ is largely pH-independent.

**Fig. 9:**
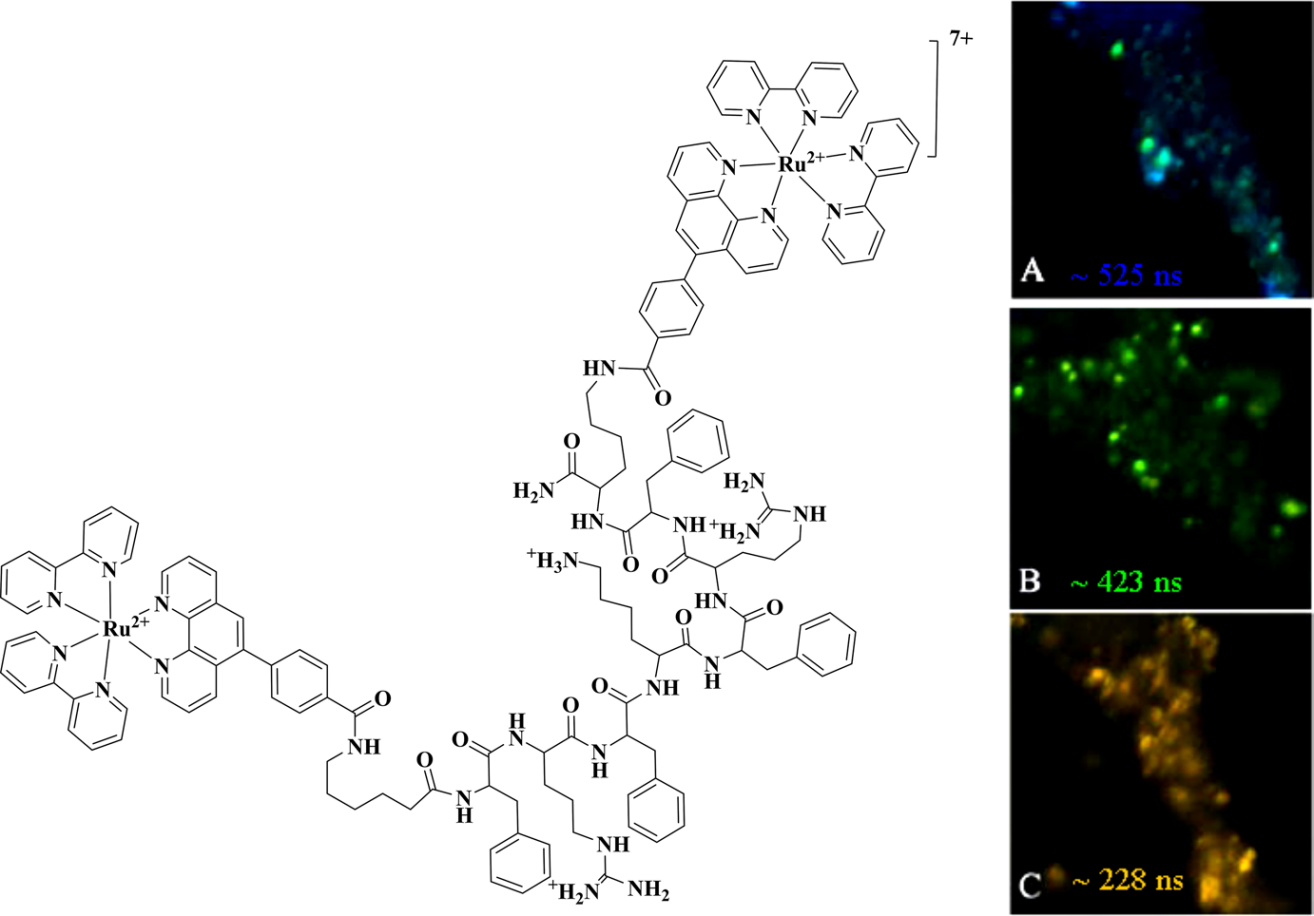
Luminescence lifetime imaging of [(Ru(bpy)_2_)_2_(phen-MPP-phen]^7+^ in live HeLa cells, in response to antimycin A treatment. PLIM was carried out following **A** incubation with Ru(II) conjugate at 70 μM for 2 h in the absence of light, and treatment with antimycin A (200 μg/ mL) for **B** 10 min and **C** 100 min [118]. Reprinted (adapted) with permission from Martin et al. [118]. Copyright 2014 American Chemical Society

The advantage of peptide vectorisation is that it may enable real-time monitoring of *local* oxygen fluctuations at a specific cellular region or organelle, and cross-reactivity may be minimised if other parameters remain unchanged while the analyte of interest is varied. For example, the mitochondrial-targeted Ru(II) conjugate reported by Keyes et al. showed dynamic response to changes in local oxygen concentrations and to elevated levels of reactive oxygen species using luminescence intensity and lifetime imaging [118]. The dinuclear ruthenium(II) probe was bridged across a mitochondrial-penetrating peptide yielding [(Ru(bpy)_2_)_2_(phen-MPP-phen]^7+^. Following exposure of HeLa cells to antimycin A, a mitochondrial uncoupler agent, PLIM studies showed that average emission lifetime of [(Ru(bpy)_2_phen-Ar)_2_-MPP]^7+^ in live HeLa cells was quenched from approximately 525–228 ns, as shown in Fig. 9.

Cross-responsiveness with other environmental factors such as pH, proteins/enzymes and lipidic environments is a key limitation of lifetime imaging for single probes in the cell environment and indeed in molecular probes in general, as it is difficult to decouple or calibrate the analytical probe and/or reference from other potential environmental influences in the complex cell matrix. However, in studies like these, where relative changes to analyte are followed rather than studies of absolute concentration, this may be less of an issue. Nanoparticle probes offer some advantages in this regard.

Nanoparticle (NP)-based systems have inherent advantages in terms of their cell permeability once they are between 50 and 200 nm and have appropriate lipophilicity and surface charge. Several NP O_2_ sensor formats have been reported; they comprise of either (1) an O_2_ probe alone or (2) a probe reference pair that generates a ratiometric signal through Förster resonance energy transfer (FRET) or (3) a probe reference pair that has independent emission signals that do not require cross-communication but can be excited at a single wavelength [241–250].

Of the latter type, our group has reported a ratiometric core–shell nanosensor, Ru-BODIPY NP, where the BODIPY reference probe was confined to a polystyrene core, offering its protection from environment, and so a stable reference signal and a ruthenium probe conjugated to the poly-lysine shell exterior allowing direct exposure to the environment and O_2_ accessibility [241]. In the Ru-BODIPY nanoparticle system, the O_2_ indicator and reference dye are spatially separated into a particle core and shell, *thus limiting any potential cross-communication*, and they are simultaneously excited at a single wavelength. The nanoparticles showed good ratiometric response to oxygen in aqueous media with a rate of quenching of 7.52 × 10^8^ M^−1^ s^1^. The emission intensity ratiometric data showed moderately good linearity (*R*^2^ = 0.9525) over a biologically relevant O_2_ range. Following surfactant-mediated uptake of RuBODIPY NPs in CHO cells, lifetime imaging studies showed that the emission lifetime of the BODIPY dye, as expected, was unaffected by the surrounding intracellular environment in contrast to the ruthenium probe, demonstrating the potential of the core–shell approach to designing new ratiometric nanotools.

Aiming to overcome the need for a membrane permeabilising agent, a ratiometric sensor was then developed where the Ru(II) oxygen sensor and reference BODIPY dye are co-encapsulated within the particle core which is permeable to oxygen, and the particle exterior is decorated solely with a poly-l-lysine shell [208]. This approach indeed permitted uptake of the self-referenced O_2_ nanoparticles in live mammalian cells, demonstrating the impact of even relatively modest surface modification of the particle on uptake. Importantly, the particles were suitable for both non-invasive hypoxia imaging using confocal microscopy (xyλ scanning) and for quantitative ratiometric intensity-based measurements of oxygen *in cellulo* using a plate reader assay. The isolation of the probe to the particle core protected it from environmental factors other than oxygen, but may impact dynamic response as the oxygen must diffuse through the particle matrix to reach the probe.

### pH

pH is an important regulator of metabolic processes in the cell and is believed to play a role in signalling. pH varies across the cell organelles, and its homeostasis may be a marker of cell health. Therefore, sensing intracellular and organelle pH is an important target analyte that has been the focus of studies in metal luminophore probes. A pH- and oxygen-sensitive iridium(III) complex was prepared by coordinating two cyclometalated ligands [2-(2, 4-difluorophenyl)pyridine; dfpp] to an Ir(III) centre along with the pic(COOH) ligand, 2-(4-carboxylphenyl)imidazo[4, 5-f][1,10]phenanthroline, carrying a terminal carboxyl moiety, thus permitting amide coupling to an octaarginine sequence in order to improve aqueous solubility [29]. The parent complex exhibited a lifetime of approximately 674 ns in degassed organic media which was reduced to 200 ns in degassed aqueous media at pH 6.9. Cytotoxicity studies showed that both the Ir(III) parent complex and conjugate were cytotoxic towards SP2 and CHO cell lines. The cytotoxic character of iridium complexes has been reported in a number of studies [69, 251], and it is likely that increased cytotoxicity compared to other transition metal luminophores is the result of its lipophilic nature inducing rapid uptake and wide distribution of the conjugate within cells.

Chao et al. reported an iridium(III) pH sensor that was coordinated to ligands containing morpholine groups [252]. They observed that morpholine promoted mitochondrial targeting, and the pH dependence of the emission intensity of the probes was explored in HeLa cells where extracellular pH was adjusted and from 6.0 to 8.0 in high-K^+^ media; equilibration with the cell interior was achieved by application of nigericin, a membrane-associating antiporter ionophore for K^+^ and H^+^. The emission intensities from the complexes within the cell were observed to respond to pH in the range of 6.0–8.0. On stimulation of apoptosis in the cells, using mitochondrial uncoupler carbonyl cyanide m-chlorophenyl hydrazine (CCCP), the emission intensity of the probes in the mitochondria was also observed to modulate; however, attributed to pH change, other quenching species may evolve in the mitochondria as a consequence of uncoupling.

As previously described, the octaarginine-driven conjugate [Ru(bpy)_2_(pic-R_8_)]^10+^ was applied as a probe for oxygen mapping using lifetime imaging [1, 88]. The emission lifetime of this complex is strongly oxygen-dependent, but the quenching constant by O_2_ is largely pH-independent, which serves the use of the probe in O_2_ mapping. Conversely, resonance Raman spectroscopy, and therefore the Raman signature signal of the probe, is strongly pH-dependent and is insensitive to O_2_, thus enabling use of the probe in pH mapping using resonance Raman spectroscopy. The probe permits multi-parameter monitoring and mapping of the intracellular environment using a single probe, single excitation and two imaging techniques enabled by the large Stokes shift of Ru(II) polypyridyl complexes. Ligands such as *pic* in this complex or *dppz* and *bpy* exhibit signature Raman signals when they participate in the MLCT the excitation is resonant with, and in the case of the ionizable pic ligand, its Raman signature grows into resonance depending on the pH of the environment/ionisation of the pic imidazole residue, thus providing a distinctive pH marker.

### Biorelevant Molecules: Receptors, Proteins, Enzymes

Lo et al. presented a series of cyclometalated Ir(III) complexes containing a perfluorobiphenyl (PFBP) moiety and their respective conjugates, afforded through reaction of PFBP with the cysteine moiety in a four amino acid sequence (FCPF, known as “π-clamp”) [155]. Following this π-clamp-mediated cysteine conjugation, novel Re(I) conjugates were prepared and applied as imaging agents but also as enzyme sensors [253].

An early study, presented by Stephenson et al., described conjugation of a rhenium complex to the peptide fMLF which is known to deliver to the formyl peptide receptor (FPR) [254]. A qualitative comparison of the cell uptake and distribution between a known FPR-targeting fluorescent probe (fluorescein-labelled fNLFNTK) and the rhenium-fMLF conjugate suggested that the rhenium probe successfully targeted the FPR.

As mentioned earlier, a rhenium(I) tricarbonyl complex was conjugated to a derivative of *T140*, a known antagonist of CXCR4 and a chemokine receptor which is overexpressed in cancer cells [177]. The rhenium conjugate was successful in sensing CXCR4, evident by a strong luminescence signal detected from cells expressing CXCR4, whereas no luminescence was detected from cells lacking the receptor.

Although rhenium peptide conjugates have been exploited as luminescent probes for interrogating different cell receptors, there has been an alternative motivation for synthesising rhenium peptide conjugates as structural analogues for “hot” technetium complex conjugates. However, in several studies, the radioactive technetium peptide conjugate was utilised to study specific cell receptors via radioimaging techniques, for instance, SPECT [255–258].

Although not exploited for its sensing capabilities, a zinc phthalocyanine complex conjugated to a receptor-targeting peptide, LARLLT, was reported to exhibit high selectivity for the epidermal growth factor (EGF) which tends to be overexpressed on the surface of cancerous cells [259]. Conjugation to the peptide increased the photodynamic efficacy and selectivity of complex against cancer cells with different receptor expression levels.

### Reactive Oxygen and Nitrogen Species (ROS/RNS)

Reactive oxygen species and reactive nitrogen species are highly reactive, often radical species, generated as part of metabolic processes within the cell. They are potentially injurious to the cell if not regulated. ROS and RNS are numerous and include species such as superoxide (O_2_^**·**−^), hydroxyl (^**·**^OH), peroxyl (RO_2_^**·**^) and alkoxyl (RO^−^) radicals nitric oxide, peroxynitrite, nitrate, nitrite and nitrogen dioxide. RNS and ROS generation pathways are inter-dependent. For example, peroxynitrite (ONOO^−^) is the product of a reaction between nitric oxide (NO) free radicals and superoxide and at abnormal levels, can induce oxidative changes in intracellular molecules, including DNA and proteins [260].

Monitoring changes in oxygen, RNS and ROS levels within the cell and particularly within the mitochondria, one of the key cellular sources of such species, is invaluable in understanding both normal physiology and disease, and also in understanding toxicity and therapeutic response.

As mentioned earlier, the mitochondria-targeting Ru(II) probe, [(Ru(bpy)_2_)_2_(phen-MPP-phen]^7+^, is capable of responding to changes in local O_2_ concentrations and also to elevated ROS levels [118].

**Fig. 10:**
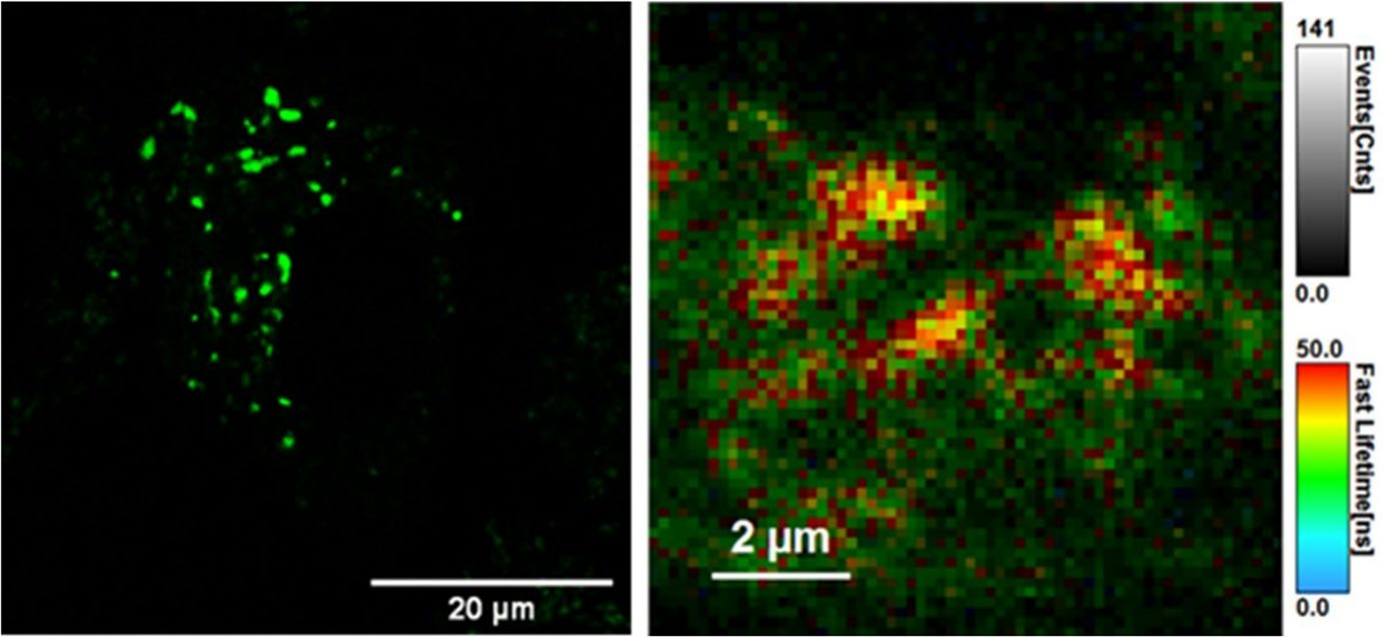
Confocal fluorescence (left) and luminescence lifetime imaging (right) of [Os-(R_4_)_2_]^10+^ confined in lysosomes at 30 μM in live A549 cells. The Os(II) conjugate exhibited lifetimes of 37 ± 1.8 ns (54%) and 9.3 ± 0.6 ns (32%). Reprinted (adapted) with permission from Ref. [10] (https://pubs.acs.org/doi/10.1021/acs.inorgchem.1c00769). Further permissions related to the material excerpted should be directed to the ACS

Recent studies have highlighted the potential of Os(II) polypyridyl complexes for detection of oxidative damage and intracellular reactive oxygen species [10, 261, 262]. The absence of oxygen sensitivity in the case of Os(II) complexes but potential redox sensitivity offers an advantage in their application as intracellular sensors over complexes of ruthenium or iridium. For example, phosphorescence lifetime imaging studies revealed that the emission lifetime of the polyarginine Os(II) conjugate, [Os-(R_4_)_2_]^10+^, was found to vary with intracellular localisation [10]. When confined to the lysosomes and surrounding cytoplasm, [Os-(R_4_)_2_]^10+^ exhibited reduced lifetimes in comparison to when it was initially taken up into the cytoplasm of cells. For example, the dominant amplitude component of the decay was measured as 92.2 ± 2.9 ns upon cytoplasmic uptake and 37 ± 1.8 ns upon lysosomal accumulation. This lifetime quenching is likely due to the presence of redox-active species as the probe luminescence was not sensitive to oxygen or pH changes [10]. Figure 10 shows the confocal and lifetime imaging of [Os-(R_4_)_2_]^10+^ upon lysosome localisation at 30 μM/48 h. Lifetime imaging studies were also carried out following uptake and accumulation of the probe within pancreatic 3D multicellular tumour spheroids, thus highlighting the suitability of such probes for monitoring metabolic changes in cells, spheroids or tissues without interference from oxygen.

The mitochondria-localised ruthenium(II) complex–cyanine (Ru-Cy5) scaffold, *although peptide-free*, is a good example of the application of such transition metal probes for *in cellulo* sensing and imaging, in this instance, of peroxynitrite in cells [263]. This energy transfer-based probe constituted a Ru(II) complex as the energy transfer donor and Cy5 as energy transfer acceptor. Following cellular uptake and mitochondrial localisation in HeLa cells, the emission of Cy5 was decreased in the presence of ONOO^−^ as a result of oxidative cleavage of the polymethine bridge which interrupts the energy transfer between Ru(II) and Cy5. The Ru-Cy5 system showed low cytotoxicity, efficient mitochondrial accumulation and good selectivity for ONOO^−^ (*over other reactive species*).

Although there are some additional examples of non-peptide metal complexes which exhibit a luminescence response to NO [264, 265] or radical species such as hypochlorite [266], there are to date limited reports of peptide metal conjugates which have been applied for monitoring intracellular redox species.

### Cell Membrane Markers

Again reflecting the versatility of Ru(II) complexes as multiparameter and multimodal imaging tools, [Ru(dppz)_2_(pic-Arg_8_)]^10+^ was used in confocal luminescence and resonance Raman imaging [109]. Owing to the dppz ligands, the complex behaved as a molecular light switch where the luminescence of the complex is extinguished in aqueous solution, *due to hydrogen bonding to the phenazine nitrogens*, but switched on in lipid vesicles. Resonance Raman intensity mapping revealed that the octaarginine conjugate crossed the membrane and distributed throughout the cell, whereas the parent complex accumulated in the cell outer membrane.

**Fig. 11:**
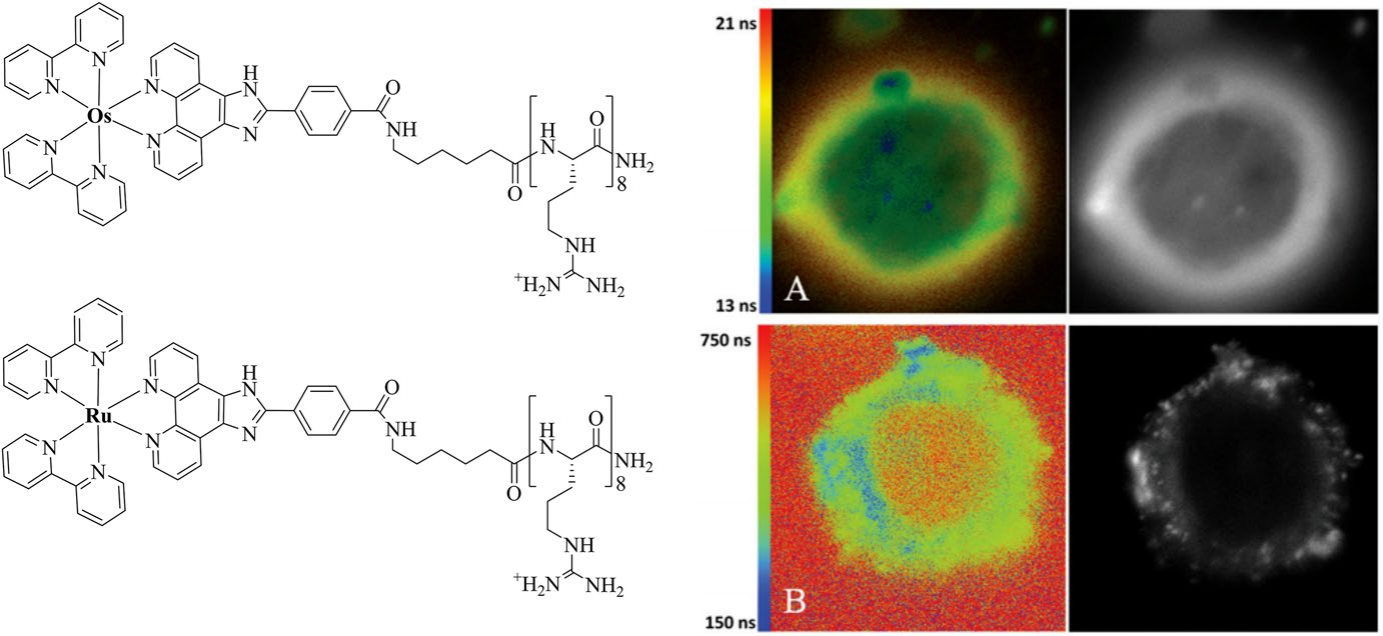
Phosphorescence lifetime imaging of **A** [Os(bpy)_2_(pic-R8)]^10+^ (70 μM/24 h) and **B** [Ru(bpy)_2_(pic-R8)]^10+^ (350 μΜ/15 min) in live SP2 cells. The false colour images and intensity mapping highlight the differences in emission lifetime of the luminophore depending on its localisation in the cell. The emission lifetime of the osmium(II) conjugate was 14.5 ± 0.5 ns in the cytoplasm and 18.8 ± 0.6 ns at the cell membrane. Reproduced from Ref. [131] with permission from the Royal Society of Chemistry

PLIM mapping of a related osmium octaarginine conjugate, [Os(bpy)_2_(pic-Arg_8_)]^10+^, revealed that the emission lifetime of the complex changed in response to the intracellular environment [131]. For example, the average lifetime was found to be 11.6 ± 0.4 ns in the cytoplasm of CHO cells and 14.5 ± 0.5 ns in SP2 cells. Additionally, a lifetime of 13 ± 1.5 ns and 18.8 ± 0.6 ns was observed for the membrane of CHO and SP2 cells, respectively. As the lifetime of the complex is oxygen-independent, this response may be due to differences in the lipid packing of the cell membrane of each cell line. As mentioned earlier, a variation in lifetime was observed for the Ru(II) analogue, [Ru(bpy)_2_(pic-Arg_8_)]^10+^, conjugate which was attributed to the increased solubility of O_2_ in the cellular membrane, thus reflecting cell membrane oxygenation [88]. Figure 11 illustrates the PLIM mapping of [Os(bpy)_2_(pic-Arg_8_)]^10+^ and [Ru(bpy)_2_(pic-Arg_8_)]^10+^ in SP2 cells.

## Conclusions

Metal complex luminophores have been widely explored as probes of biological molecules, particularly of DNA in its variety of motifs, since the 1980s. In the past decade, their putative application as probes within the cellular environment has been realised, and metal complex luminophores are now rapidly extending beyond the scope of in vitro studies to diverse applications in cellular imaging, intracellular sensing and theranostics.

The beauty of metal complexes is that they are highly synthetically versatile and can be tailored to their application by tuning their photophysical properties (e.g*., molecular brightness, NIR emission*), through modification of the metal centre and/or the coordinated ligands, through relatively facile synthetic methods. They can be tailored to meet the demands of the technique used for imaging or sensing whether that is phosphorescence lifetime imaging microscopy, luminescence intensity-based sensing using a plate reader or vibrational imaging such as Raman mapping.

Critically, metal complex luminophores can be readily conjugated to vectorising functionalities to facilitate cellular uptake and intracellular targeting. Peptides are a valuable means of promoting such permeation and targeting within cells, and their efficacy in this regard has been known for many years in drug delivery. As signal molecules, they can drive their cargo very precisely within the cell. Peptides can confer improved solubility and lower cytotoxicity on their cargo and can be synthesised readily via high throughput and often automated solid-state synthesisers. Conjugation of metal complexes to cell-penetrating and signal peptides has been used for the efficient delivery of complexes in cell monolayers, but also recently in multicellular spheroids. Peptides, to date, have been very effective in driving complex cargo to the cell and targeting, but the mechanism is not fully elucidated; for example, the role of the counter-anions accompanying the charged conjugate seem to have a strong effect on membrane permeability, but remains to be fully explored and exploited. The possibilities are extensive, and metal complex luminophore peptide conjugates are likely to find increasing application in theranostic applications.

Although peptides can promote fairly rapid cellular uptake (i.e. < 1–24 h) required for imaging, the application of the metallo-peptide conjugates in bioassay experiments has been less explored. In terms of the metal complex photophysics, key future challenges lie in maximising probe brightness, as inorganic luminophores do not compete well with organic fluorophores in this regard, but maximising the absorbance cross section and tuning analytically relevant responsive luminescence will ensure that even if they do not match organic probes in terms of emission quantum yield, they offer versatile alternatives.

Another challenge is the *in cellulo* limit of detection and limit of quantification of the species that the probe is sensing. The influence of the incredibly complex cytosol composition or organelle environment complicates photophysical effects and sensing capability.

Metal complex luminophores translate well to confocal luminescence imaging techniques that permit focused interrogation of a specific cell region to yield site-specific biorelevant information with good optical sectioning and indeed look likely to provide important solutions in super-resolution methods too. In particular, lifetime imaging is the method of choice for visualising spatial oxygen distribution in cells and particularly in multicellular spheroid samples. However, metal complex luminophores are less widely tested in the more common and conventional methods used in biological labs. In particular, as probes for plate reader-based bioassays in 96-well format which are a widely used and high-throughput approach where emission, lifetime or absorbance is collected from multiple cells across a well format. Highly selective probe targeting or switchable probes would be required with limited contribution from emission of the complex outside the region of interest for such applications. The targeting ability of the conjugate dye would also have to be extremely precise. Few of the dyes described above meet this criterion of highly specific localisation that would be required for a well format assay yet. Nonetheless, they have proven to be effective imaging probes for investigating cellular membrane dynamics, sensing receptor expression and monitoring redox species in mitochondria, pH fluctuations and DNA interactions under imaging conditions *in cellulo* and look likely with further advances in precision targeting to emerge as powerful tools for diverse bioanalysis.

## Abbreviations

3D: Three-dimensional
CPP: Cell-penetrating peptide
DAPI: 4′, 6-Diamidino-2-phenylindole
DLS: Dynamic light scattering
dpp or dip: 4, 7-Diphenyl-1, 10-phenanthroline
Dppz: Dipyrido[3, 2-a:2′, 3′-c]phenazine
FLIM: Fluorescence lifetime imaging
icO_2_: Intracellular oxygen
IP: Imidazo[4, 5-f][1, 10]-phenanthroline
MLCT: Metal-to-ligand charge transfer
MMP: Mitochondrial membrane potential
MPP: Mitochondria-penetrating peptide (FrFKFrFK)
phen: 1,10-Phenanthroline
PLIM: Phosphorescence lifetime imaging microscopy
STED: Stimulated emission depletion microscopy
TAP: 1,4,5,8-tetraazaphenanthrene
